# Maternal Nanos inhibits Importin-α2/Pendulin-dependent nuclear import to prevent somatic gene expression in the *Drosophila* germline

**DOI:** 10.1371/journal.pgen.1008090

**Published:** 2019-05-15

**Authors:** Miho Asaoka, Kazuko Hanyu-Nakamura, Akira Nakamura, Satoru Kobayashi

**Affiliations:** 1 Life Science Center for Survival Dynamics, Tsukuba Advanced Research Alliance, University of Tsukuba, Tsukuba, Ibaraki, Japan; 2 Department of Germline Development, Institute of Molecular Embryology and Genetics, Kumamoto University, Kumamoto, Japan; 3 Graduate School of Pharmaceutical Sciences, Kumamoto University, Kumamoto, Japan; 4 Graduate School of Life and Environmental Sciences, University of Tsukuba, Tsukuba, Ibaraki, Japan; College de France CNRS, FRANCE

## Abstract

Repression of somatic gene expression in germline progenitors is one of the critical mechanisms involved in establishing the germ/soma dichotomy. In *Drosophila*, the maternal Nanos (Nos) and Polar granule component (Pgc) proteins are required for repression of somatic gene expression in the primordial germ cells, or pole cells. Pgc suppresses RNA polymerase II-dependent global transcription in pole cells, but it remains unclear how Nos represses somatic gene expression. Here, we show that Nos represses somatic gene expression by inhibiting translation of maternal *importin-α2* (*impα2*) mRNA. Mis-expression of Impα2 caused aberrant nuclear import of a transcriptional activator, Ftz-F1, which in turn activated a somatic gene, *fushi tarazu* (*ftz*), in pole cells when Pgc-dependent transcriptional repression was impaired. Because *ftz* expression was not fully activated in pole cells in the absence of either Nos or Pgc, we propose that Nos-dependent repression of nuclear import of transcriptional activator(s) and Pgc-dependent suppression of global transcription act as a ‘double-lock’ mechanism to inhibit somatic gene expression in germline progenitors.

## Author summary

Identification of the molecular mechanism underlying germline segregation from the soma is a fundamental goal of reproductive, cellular, and developmental biology. In many animal species, repression of somatic gene expression in germline progenitors is critical for the germ/soma segregation. In *Drosophila*, germ plasm, a specialized ooplasm partitioned into germline progenitors, contains maternal factors sufficient to repress somatic differentiation. Here, we show that a subset of somatic genes is derepressed when two maternal factors, Nanos (Nos) and Polar granule component (Pgc) are concomitantly suppressed. While Pgc is known to suppress RNA polymerase II (Pol II) activity, how Nos achieves this effect remains obscure. We find that Nos represses production of Importin-α2 that is essential for nuclear import of transcriptional activators for somatic gene expression in germline progenitors. Thus, we propose that Nos-dependent inhibition of nuclear import of transcriptional activators and Pgc-dependent suppression of Pol II activity acts as a ‘double-lock’ mechanism to ensure tight inhibition of somatic gene expression in germline progenitors. Since Nos is evolutionarily conserved, and a transient suppression of Pol II is a trait of germline progenitors of diverse animal species, the ‘double-lock’ mechanism may play a widespread role in germ/soma segregation.

## Introduction

How germ cell fate is established and maintained is a century-old question in developmental, cellular, and reproductive biology. Metazoan species have two distinct modes of germline specification [1]. In some species, germline progenitors are characterized by inheritance of a specialized ooplasm, or the germ plasm, which contains maternal factors necessary and sufficient for germline development [2–7]. In other species, germline progenitors are specified by inductive signals from surrounding tissues [8, 9]. Irrespective of the mode of germline specification, transcriptional repression of somatic genes is common in germline progenitors [10–16], implying that this phenomenon is critical for separation of the germline from the soma.

In *Drosophila*, the germ plasm is localized in the posterior pole of cleavage embryos (stage 1–2), and is partitioned into germline progenitors called pole cells (stage 3–4). In pole cells of blastoderm embryos (stage 4–5), the genes required for somatic differentiation are transcriptionally repressed by two maternal proteins in the germ plasm, Polar granule component (Pgc) and Nanos (Nos) [10, 15, 17]. Pgc is a *Drosophila*-specific peptide that suppresses RNA polymerase II-dependent transcription in pole cells by inhibiting positive transcriptional elongation factor b (P-TEFb) function [17]. By contrast, Nos is an evolutionarily conserved protein that plays an essential role in germline development in various animals [18]. For example, in *Drosophila*, pole cells lacking Nos (*nos* pole cells) can adopt a somatic, rather than a germline, fate [19]. Furthermore, depletion of Nos is reported to show ectopic expression of somatic genes, such as *fushi tarazu* (*ftz*), *even-skipped* (*eve*), and the sex-determination gene *Sex lethal (Sxl)*, in pole cells [15]. Thus, maternal Nos is required in pole cells for repression of somatic genes and establishment of the germ/soma dichotomy. However, the mechanism by which Nos represses somatic gene expression remains unknown.

Nos acts as a translational repressor of mRNAs that harbor a discrete sequence motif called Nanos Response Element (NRE) in the 3´ UTR. NRE contains an evolutionarily conserved Pumilio (Pum)-binding sequence, UGU trinucleotide [20–22]. In abdominal patterning, Pum represses translation of maternal *hunchback (hb)* mRNA by binding to NREs in its 3´ UTR and recruiting Nos to the RNA/protein complex [23, 24]. Deletion of the NREs from *hb* mRNA causes its ectopic translation in the posterior half of embryos, which in turn suppresses abdomen formation [25, 26]. Furthermore, deletion of NREs causes *hb* translation in pole cells [25, 26], suggesting that NRE-dependent translational repression occurs in pole cells. Indeed, Nos represses translation of *head involution defective (hid)* mRNA in pole cells in an NRE-like-sequence-dependent manner [27]. In addition, Nos and Pum repress *Cyclin B* translation in pole cells by binding to a discrete sequence containing two UGU trinucleotides (*Cyclin B* NRE) [26]. These findings led us to speculate that Nos, along with Pum, represses somatic gene expression in pole cells by suppressing translation of mRNAs containing NRE or UGU in their 3´ UTRs.

Here, we report that, in pole cells, Nos, along with Pum, represses translation of *importin-α2* (*impα2*)*/Pendulin/oho31/CG4799* mRNA, which contains an NRE-like sequence in its 3´ UTR [28]. The *impα2* mRNA encodes a *Drosophila* Importin-α homologue that plays a critical role in nuclear import of karyophilic proteins [28–31]. Nos inhibits expression of a somatic gene, *ftz*, in pole cells by repressing Impα*2*-dependent nuclear import of the transcriptional activator, Ftz-F1. Based on our observations, we propose that Nos-dependent inhibition of nuclear import of transcriptional activators and Pgc-dependent global transcriptional silencing act as a ‘double-lock’ mechanism to repress somatic gene expression in pole cells.

## Results and discussion

### Nos, along with Pum, represses production of Impα2 in pole cells

**Fig 1:**
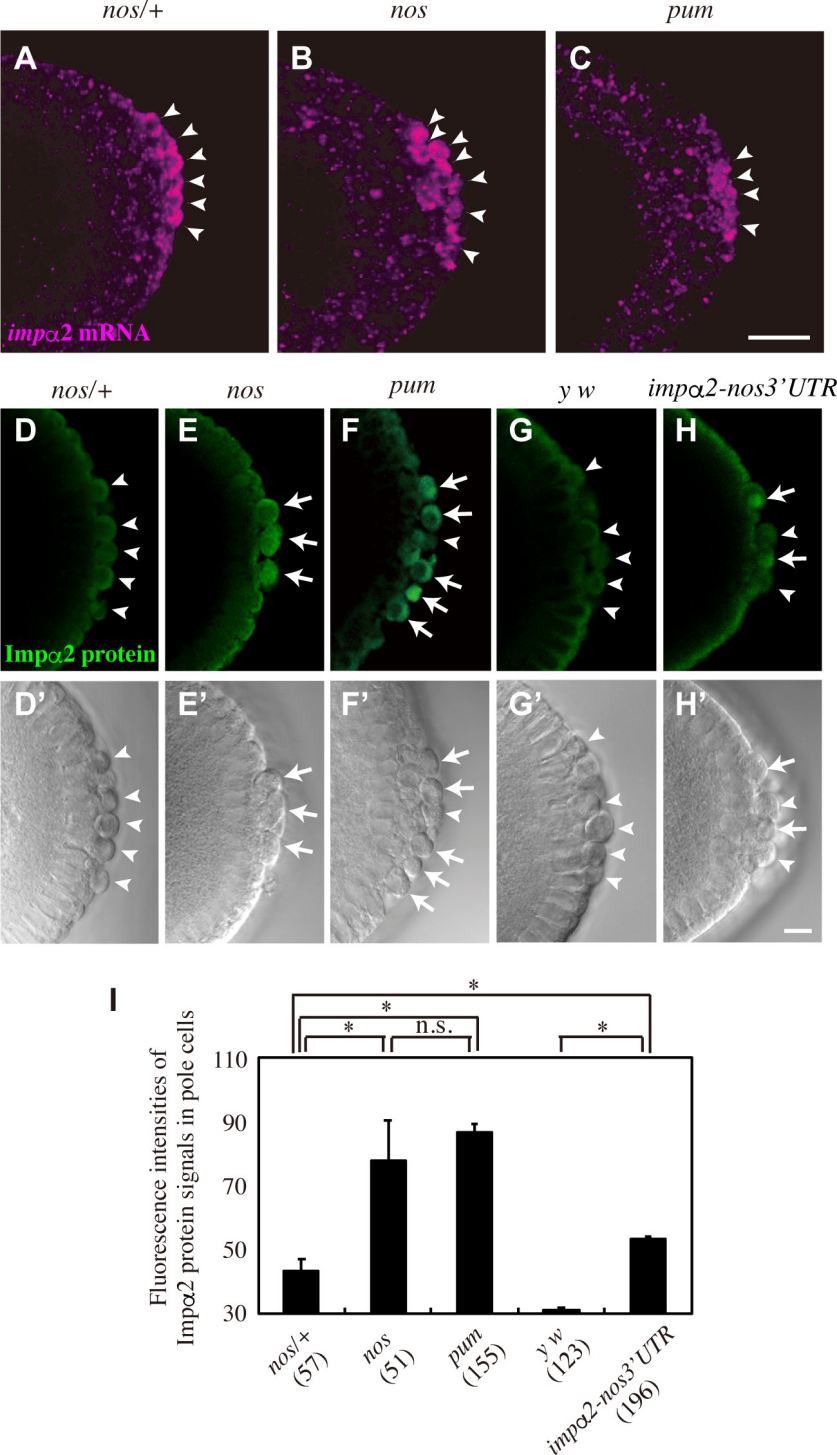
Nos and Pum repress Impα2 production in pole cells. (**A–C**) *impα2* mRNA expression in pole cells of embryos derived from *nos*/+ (**A**), *nos/nos* (*nos*) (**B**), and *pum/pum* (*pum*) females (**C**) mated with *y w* males. Stage-5 embryos were stained for *impα2* mRNA. Arrowheads point to pole cells. (**D–H**, **D’–H’**) Impα2 protein expression in pole cells of embryos derived from *nos/*+ (**D and D’**), *nos* (**E** and **E’**), and *pum* females (**F** and **F’**), and *y w* females with (*impα2-nos3´UTR*) (**H** and **H’**) or without two copies of *impα2-nos3´UTR* (*y w*) (**G** and **G’**). Stage-5 embryos were stained with anti-Impα2 23aa antibody (green, **D–H**), which recognizes only Impα2 protein among the Importin-α family of proteins [48]. DIC images (**D’–H’**) are also shown. Arrows and arrowheads point to pole cells with and without Impα2 expression, respectively. Scale bars, 20 μm (**C**) and 10 μm (**H’**). (**I**) Fluorescence intensities of Impα2 protein signals in pole cells of embryos derived from *nos*/+, *nos*, *pum*, *y w*, and *impα2-nos3´UTR* females. Embryos from late stage 4 to stage 6 were stained with anti–Impα2 23aa antibody, and fluorescence intensities of Impα2 signals were measured (see Materials and Methods). Mean values of fluorescence intensities (± SE) are shown. For each genotype, 7–17 embryos were examined. The numbers of pole cells measured are shown in parentheses. Significance was calculated using paired t-test (*: *P* < 0.01, n.s.: P > 0. 05).

**Fig 2:**
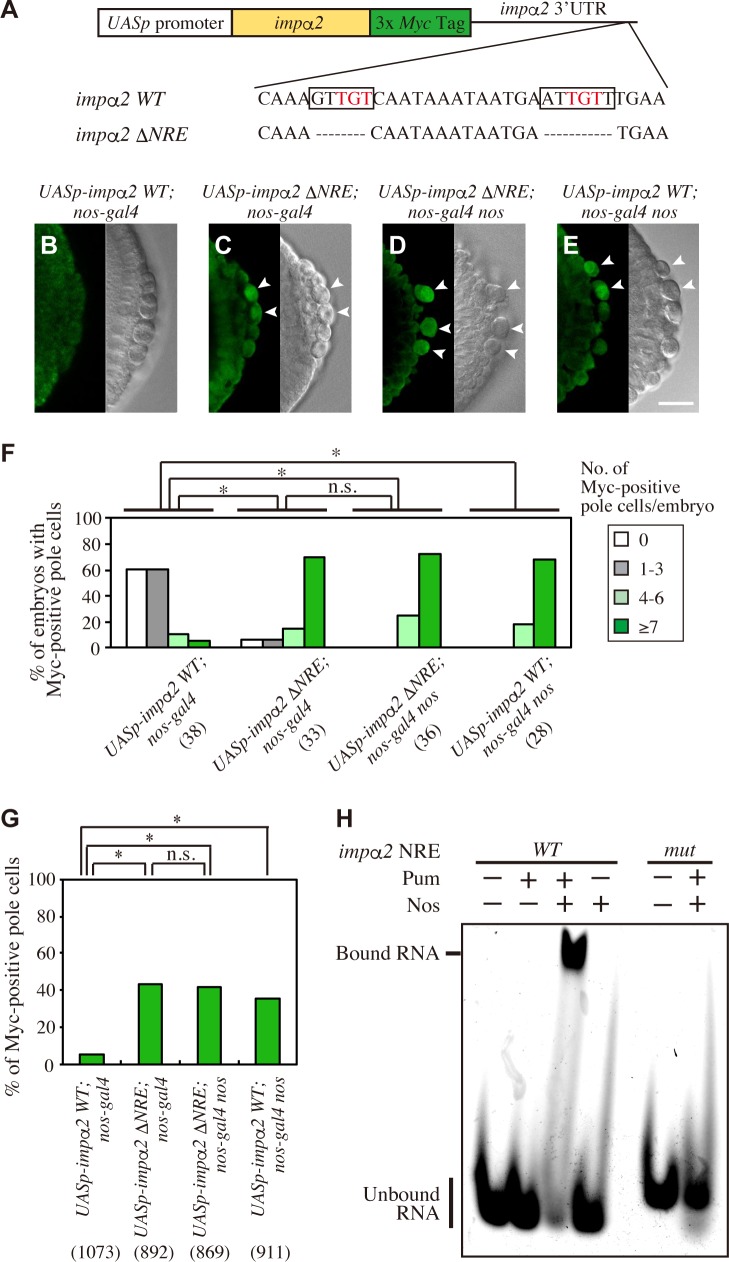
Nos represses *impα2* translation in an NRE-like sequence–dependent manner. (**A**) Schematic representation of *UASp-impα2 WT* and *UASp-impα2* Δ*NRE*, which express *impα2 WT* and *impα2 ΔNRE* mRNAs, respectively. A triple *Myc* tag sequence (green) was inserted just before the termination codon in the *impα2* protein-coding region (yellow). *impα2 WT* mRNA retains an intact 3´ UTR of *impα2* containing a single NRE-like sequence, GUUGU(Xn)AUUGUU (boxed) [28]. By contrast, *impα2 ΔNRE* contains an altered *impα2* 3´ UTR, in which the sequences GUUGU and AUUGUU were precisely deleted. Evolutionarily conserved Pum-binding sequences (UGU trinucleotides) are shown in red [20–22]. (**B–E**) Stage-5 embryos derived from *UASp-impα2 WT/+; nos-gal4/+* (*UASp-impα2 WT; nos-gal4*) (**B**), *UASp-impα2 ΔNRE/+; nos-gal4/+* (*UASp-impα2 ΔNRE; nos-gal4*) (**C**), *UASp-impα2 ΔNRE/+; nos-gal4 nos*/*nos* (*UASp-impα2 ΔNRE; nos-gal4 nos*) (**D**), and *UASp-impα2 WT/+; nos-gal4 nos*/*nos* (*UASp-impα2 WT; nos-gal4 nos*) females (**E**) mated with *y w* males were stained for Myc (green). DIC images (right) are also shown. Arrowheads point to pole cells expressing Myc-tagged protein. Scale bar, 20 μm. (**F and G**) Expression of Myc was examined in pole cells of embryos from late stage 4 to stage 6. Embryos were derived from females described above. Percentages of embryos carrying 0 (white), 1–3 (gray), 4–6 (pale green), or ≥7 (green) pole cells with Myc signal are shown in **F**. Percentages of pole cells with Myc signal are shown in **G**. The numbers of embryos or pole cells examined are shown in parentheses. Significance was calculated using Fisher’s exact test (*: P < 0.01, n.s.: P > 0.1). (**H**) EMSA was performed using *impα2* RNA fragment containing wild-type (*WT*) or mutated (*mut*) NRE-like sequence; nucleotide sequences are shown in S2 Fig. Labeled RNA with (+) or without (-) Pum or Nos was incubated as described in Materials and Methods.

Maternally supplied *impα2* mRNA is distributed throughout cleavage embryos. When embryos develop to the blastoderm stage, *impα2* mRNA is degraded in the somatic region, but not in pole cells, resulting in enrichment of *impα2* mRNA in pole cells [28] (Fig 1A). However, we found that expression of Impα2 protein was at background levels in pole cells [28] (Fig 1D and 1G). Because *impα2* mRNA contains a sequence very similar to the NRE (hereafter, NRE-like sequence) in its 3´ UTR [25, 28] (Fig 2A), we assumed that *impα2* mRNA is a target of Nos/Pum-dependent translational repression in pole cells. To investigate this possibility, we first monitored the expression of the Impα2 protein in pole cells of embryos lacking maternal Nos or Pum (*nos* or *pum* embryos, respectively). In these pole cells, expression of Impα2 protein was higher than in those of control (*nos*/+) embryos (Fig 1D–1F and 1I and S1 Fig). Because neither *nos* nor *pum* mutation affected the *impα2* mRNA level in pole cells (Fig 1B and 1C), these observations show that Nos and Pum repress protein expression from the *impα2* mRNA in pole cells.

We next investigated whether this repression is mediated by the NRE-like sequence in the *impα2* 3´ UTR. To this end, *impα2* mRNA, with or without the NRE-like sequence (*impα2 WT* and *impα2 ΔNRE*, respectively) (Fig 2A), was maternally supplied to embryos, and their protein expression was examined in pole cells at the blastoderm stage. Because a triple Myc tag sequence was inserted at the C-terminal end of the coding sequence, protein expression from these mRNAs could be monitored using an anti-Myc antibody. When *impα2 WT* mRNA was supplied to normal (*y w*) embryos, the tagged protein was expressed at low levels in the soma, but was barely detectable in pole cells (Fig 2B, 2F and 2G). By contrast, the tagged protein from *impα2 ΔNRE* mRNA was detected in normal pole cells (Fig 2C, 2F and 2G). Similar protein expression was observed in pole cells lacking Nos (*nos* pole cells), when *impα2 WT* mRNA was supplied (Fig 2E, 2F and 2G), as well as when *impα2 ΔNRE* mRNA was supplied (Fig 2D, 2F and 2G). Because the frequency of tagged protein expression from *impα2 ΔNRE* mRNA did not increase in cells lacking Nos (Fig 2F and 2G), these results indicate that the NRE-like sequence mediates Nos-dependent repression of Impα2 protein expression in pole cells.

The NRE-like sequence of *impα2* mRNA contains two UGU trinucleotides (Fig 2A). The UGU trinucleotide is a core sequence of an RNA motif (Nos-Pum SEQRS motif: 5´-HWWDUGUR) that was highly enriched in a SEQRS (*in vitro* selection, high-throughput sequencing of RNA, and sequence specificity landscapes) analysis of the Nos–Pum–RNA ternary complex (Fig 7 in the article [22]). Hence, we asked whether Pum and Nos form a ternary complex with *impα2* mRNA in an NRE-like sequence–dependent manner. To address this question, we performed electrophoretic mobility shift assay (EMSA) using the Pum RNA-binding domain and the Nos protein containing Zn finger motifs and C-terminal region, which are reported to form a Nos–Pum–target RNA ternary complex *in vitro* [22]. We found that Nos and Pum together, but neither alone, formed a complex with *impα2* RNA containing an NRE-like sequence (*WT*) (Fig 2H and S2 Fig), whereas alteration of the NRE-like sequence (*mut*) (S2 Fig) abolished this interaction (Fig 2H). These results demonstrate that Nos and Pum are able to interact with the *impα2* 3´ UTR in an NRE-like sequence–dependent manner. The observations described above led us to conclude that Nos, along with Pum, directly represses *impα2* translation in pole cells.

### Nos suppresses nuclear import of a transcription factor, Ftz-F1, by repressing Impα2

**Fig 3:**
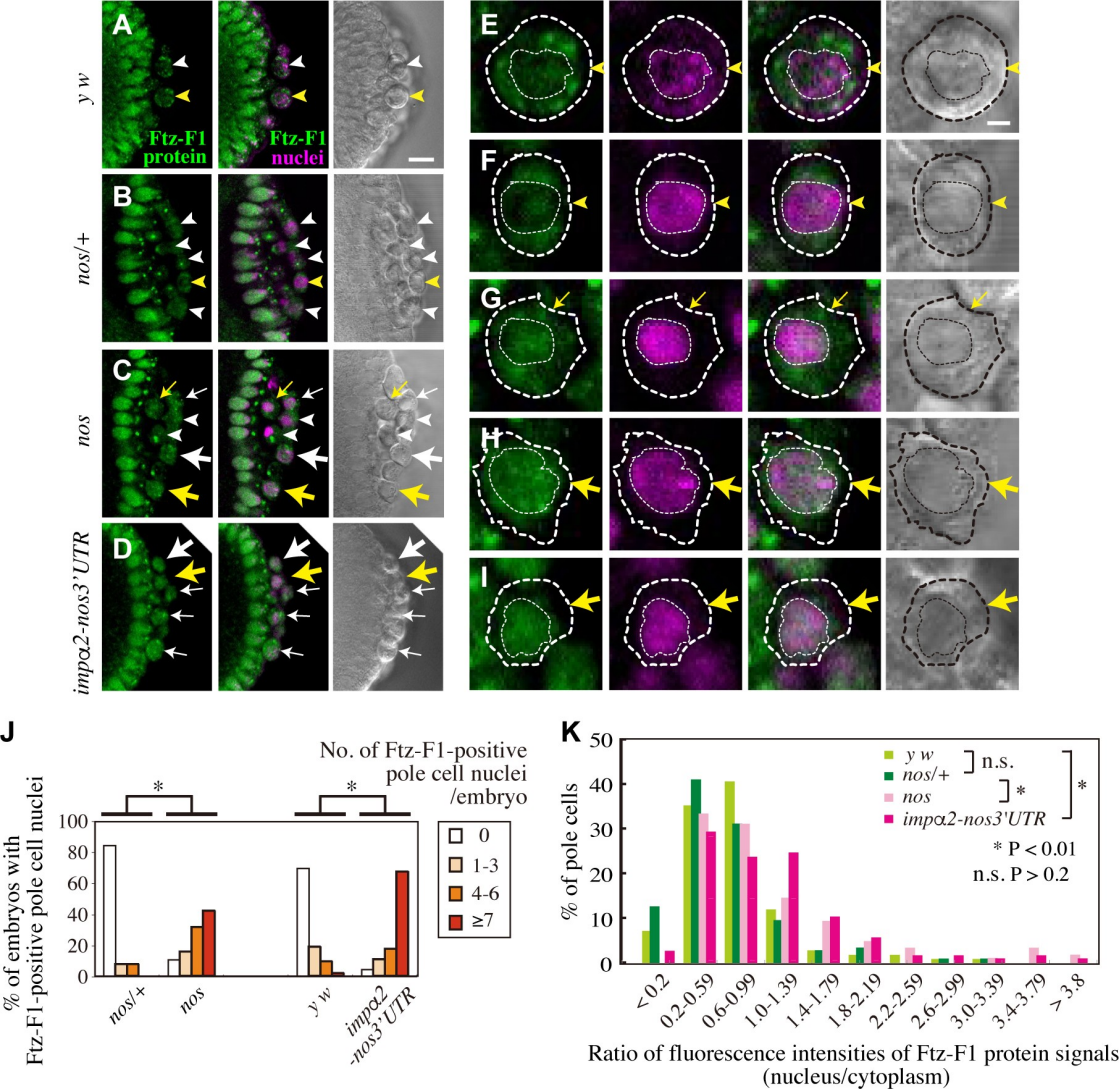
Nos represses nuclear import of Ftz-F1 in pole cells by inhibiting Impα2 production. (**A–D**) Ftz-F1 distribution in pole cells of embryos derived from *y w* (**A**), *nos*/+ (**B**), *nos* (**C**), and *impα2-nos3´UTR* (**D**) females mated with *y w* males. Stage-5 embryos were double-stained for Ftz-F1 (green) and nuclei (propidium iodide: magenta). DIC images (right) are also shown. Large arrows and arrowheads point to pole cells with Ftz-F1 signal enriched in the nucleus and cytoplasm, respectively. Small arrows point to pole cells with Ftz-F1 signal evenly distributed in the nucleus and cytoplasm. Note that Ftz-F1 is enriched in somatic nuclei. (**E–I**) Magnified images of pole cells double-stained for Ftz-F1 (green) and propidium iodide (magenta). Pole cells shown by yellow arrowheads in **A** (**E**) and **B** (**F**), small and large yellow arrows in **C** (**G** and **H**), and large yellow arrow in **D** (**I**) are shown. DIC images (right) are also shown. Dashed thick and thin lines outline pole cells and their nuclei, respectively. Scale bars, 10 μm (**A**) and 2 μm (**E**). (**J**) Expression of Ftz-F1 was examined in pole cell nuclei of embryos from late stage 4 to stage 6. Embryos were derived from *nos/+*, *nos*, *y w*, and *impα2-nos3´UTR* females mated with *y w* males. Percentages of embryos containing 0 (white), 1–3 (pale orange), 4–6 (orange), or ≥7 (red) pole cells with enrichment of Ftz-F1 signal in the nucleus are shown. For each genotype, 19–57 embryos were observed. Significance was calculated using Fisher’s exact test (*: *P* < 0.01). (**K**) Nuclear import of Ftz-F1 in pole cells of embryos derived from *y w* (lime green), *nos/+* (green), *nos* (pink), and *impα2-nos3´UTR* females (pinkish-purple), mated with *y w* males. Embryos from late stage 4 to stage 5 were double-stained with anti-Ftz-F1 antibody and DAPI or propidium iodide. Fluorescence intensities of Ftz-F1 signal in the nuclear and cytoplasmic areas of individual pole cells were measured on each section of serial confocal images, and the ratio of fluorescence intensities (nucleus/cytoplasm) was calculated (see Materials and Methods). Percentages of pole cells with each fluorescence intensity ratio are shown. For each genotype, 133–299 pole cells were counted. Significances were calculated using chi-square test.

Impα2 is a *Drosophila* homologue of Importin-α that mediates nuclear import of karyophilic proteins with classical nuclear localization signal (NLS) [28–31]. We predicted that ectopic production of Impα2 in *nos* pole cells would cause aberrant nuclear import of NLS-containing karyophilic proteins. To explore this possibility, we focused on a transcriptional activator, Ftz-F1, which contains a classical NLS and is expressed throughout early embryos, including pole cells [32–34]. In normal embryos, Ftz-F1 was enriched in the cytoplasm of pole cells, although it was in the nuclei of somatic cells (Fig 3A, 3B, 3E, 3F, 3J and 3K). In the absence of maternal Nos, the percentage of embryos with Ftz-F1 signal accumulating in pole-cell nuclei was higher than in normal embryos (Fig 3C, 3G, 3H and 3J). Furthermore, the nuclear/cytoplasmic ratio of Ftz-F1 signal intensities in *nos* pole cells was higher than in normal pole cells (Fig 3G, 3H and 3K). To determine whether this aberrant concentration of Ftz-F1 was caused by mis-expression of Impα2, we expressed Impα2 ectopically in pole cells of normal embryos (Fig 1H and 1I). To this end, *impα2* mRNA in which the 3´ UTR was replaced with the *nos* 3´ UTR, was maternally supplied under the control of the *nos* promoter; the mRNA was localized to the germ plasm and pole cells under the control of the *nos* 3´ UTR [35, 36]. The percentage of these embryos (*impα2-nos3´UTR* embryos) with Ftz-F1 in pole-cell nuclei and the nuclear/cytoplasmic ratio of Ftz-F1 intensities in their pole cells were higher than those of normal pole cells (Fig 3D, 3I, 3J and 3K). These observations suggest that mis-expression of Impα2 in pole cells caused by depletion of maternal Nos results in aberrant nuclear import of Ftz-F1.

### Mis-expression of Impα2 in the absence of Pgc function results in ectopic expression of somatic genes in pole cells

**Fig 4:**
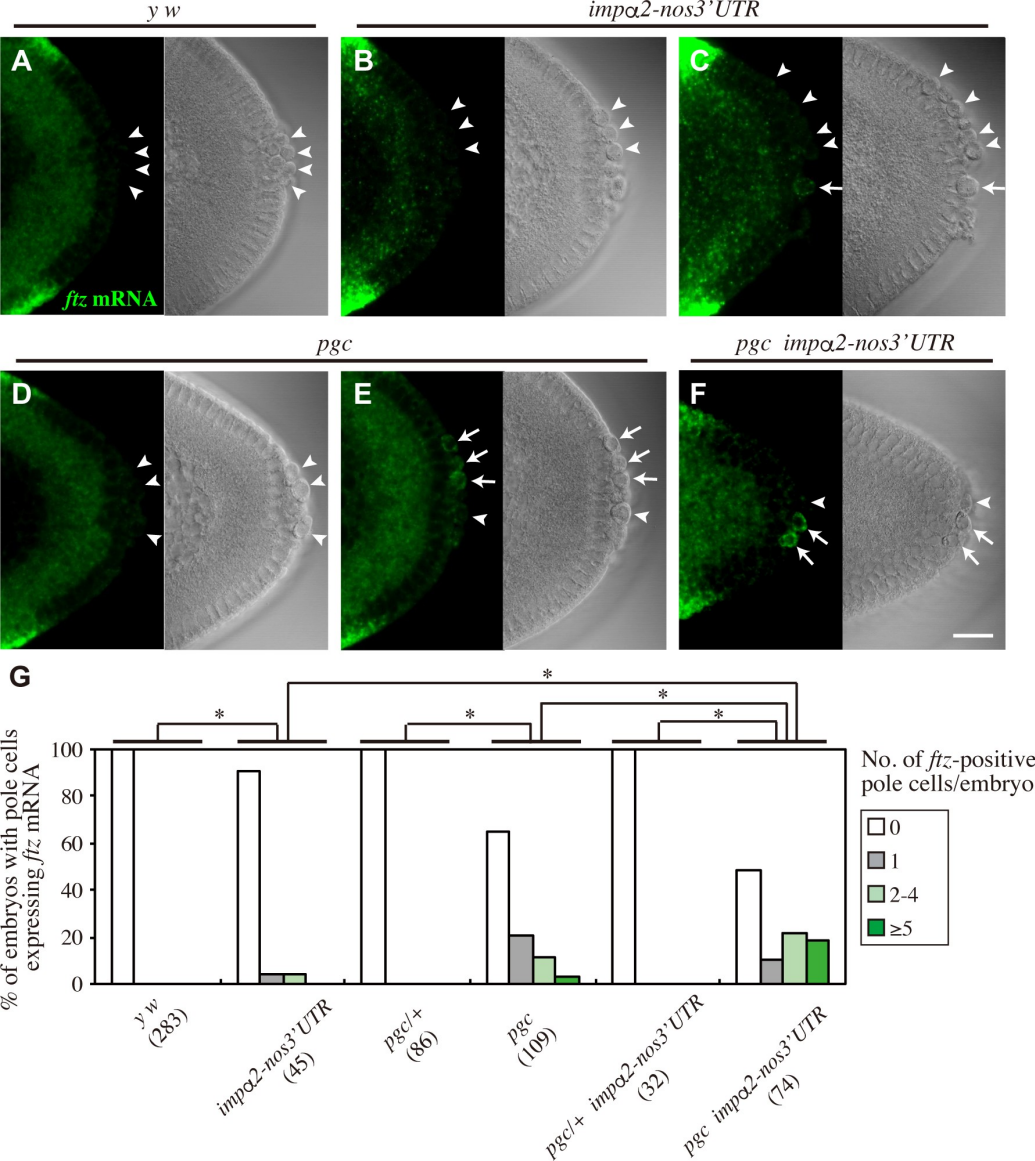
Mis-expression of Impα2 causes ectopic expression of *ftz* in pole cells lacking Pgc. (**A–F**) *ftz* mRNA expression in pole cells of embryos derived from *y w* (**A**) and *impα2-nos3´UTR* females (**B** and **C**), and from *pgc/pgc* females with (*pgc impα2-nos3´UTR*) (**F**) or without two copies of *impα2-nos3´UTR* (*pgc*) (**D** and **E**). Stage-5 embryos were stained for *ftz* mRNA (green, left). DIC images (right) are also shown. Arrows or arrowheads point to pole cells with or without *ftz* signal, respectively. Although *ftz* signal was occasionally detected in pole cells of *impα2-nos3´UTR* embryos (**C**) and *pgc* embryos (**E**), the signal intensity in these pole cells was usually less than that in pole cells of *pgc impα2-nos3´UTR* embryos (**F**). Scale bar, 20 μm. (**G**) Expression of *ftz* mRNA was examined in pole cells of embryos from late stage 4 to stage 5. Embryos were derived from *y w*, *impα2-nos3´UTR*, *pgc/+*, *pgc*, *pgc/+*; *impα2-nos3´UTR/impα2-nos3´UTR* (*pgc/+ impα2-nos3´UTR*), and *pgc impα2-nos3´UTR* females mated with *y w* males. Percentages of embryos containing 0 (white), 1 (gray), 2–4 (pale green), or ≥5 (green) pole cells with *ftz* mRNA signal are shown. The numbers of embryos examined are shown in parentheses. Significance was calculated using Fisher’s exact test (*: *P* < 0.01).

**Fig 5:**
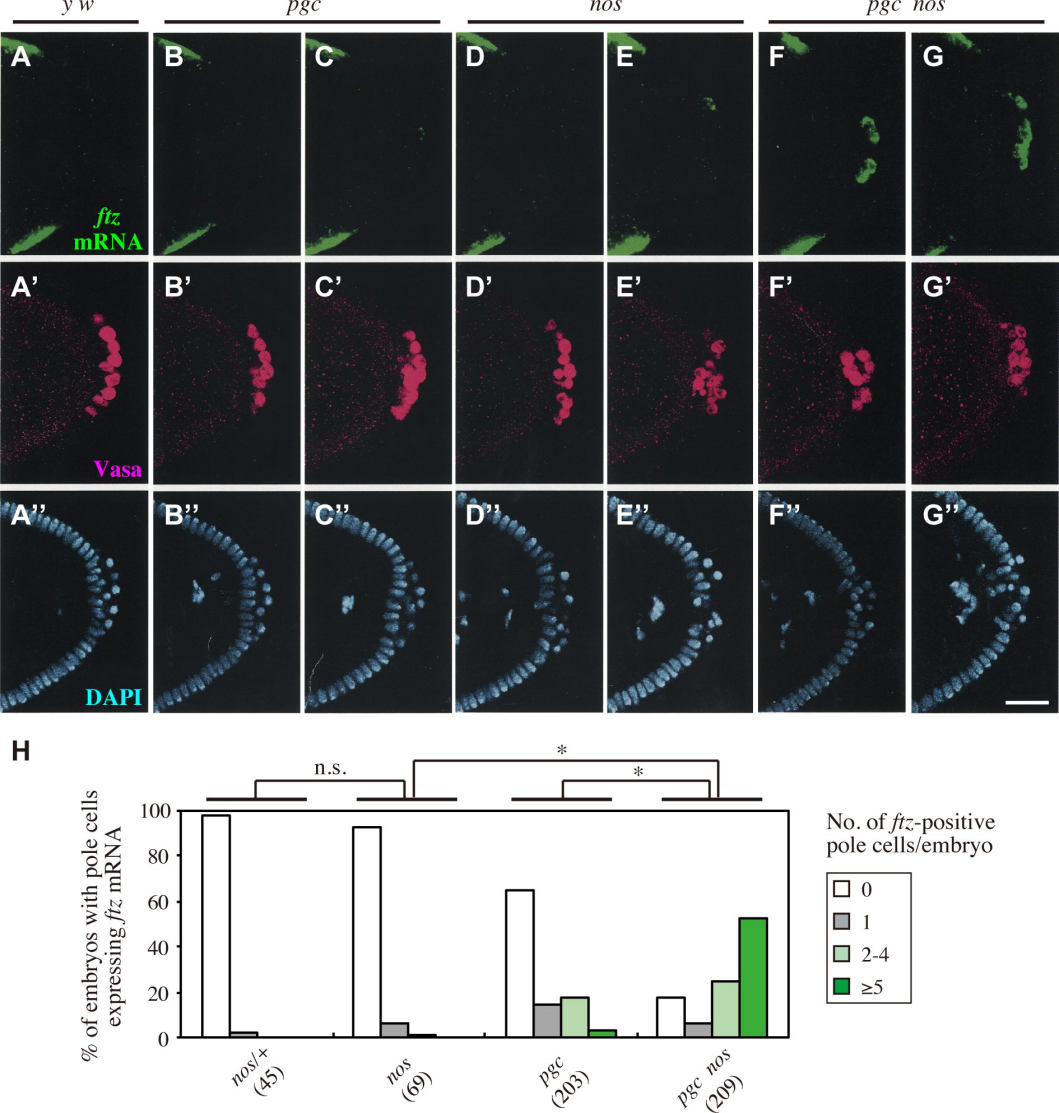
Nos and Pgc are both required to repress *ftz* expression in pole cells. (**A–G**, **A’–G’**, **A”–G”**) *ftz* mRNA expression in pole cells of embryos derived from *y w* (**A–A”**), *pgc/Df* (*pgc*) (**B–B”** and **C–C”**), *nos* (**D–D”** and **E–E”**), and *pgc/pgc*; *nos/nos* (*pgc nos*) females (**F–F”** and **G–G”**) mated with *y w* males. Stage-5 embryos were triple-stained for *ftz* mRNA (green), Vasa (magenta), and nuclear DNA (DAPI: blue). Scale bar, 30 μm. (**H**) Expression of *ftz* mRNA was examined in pole cells of embryos from late stage 4 to stage 5. Embryos were derived from *nos/+*, *nos*, *pgc*, and *pgc nos* females mated with *y w* males. Percentages of embryos containing 0 (white), 1 (gray), 2–4 (pale green), or ≥5 (green) pole cells with *ftz* mRNA signal are shown. The numbers of embryos examined are shown in parentheses. Significance was calculated using Fisher’s exact test (*: *P* < 0.01, n.s.: *P* > 0.5).

Depletion of maternal Nos results in ectopic expression of the somatic genes *ftz*, *eve* and *Sxl* in pole cells [15]. Because Ftz-F1 is required for proper expression of *ftz* in the soma [37–41], we asked whether mis-expression of Impα2 causes ectopic expression of *ftz* in pole cells. In normal embryos, *ftz* mRNA was expressed in seven stripes of somatic cells [42], but never expressed in pole cells [percentage of embryos expressing *ftz* in pole cells (pe) = 0%; number of embryos examined (n) = 283] (Fig 4A and 4G). By contrast, in *impα2-nos3´UTR* embryos, *ftz* mRNA was rarely detectable in pole cells (pe = 8.9%, n = 45) (Fig 4B, 4C and 4G). We assumed that this low frequency of *ftz* expression was due to Pgc-mediated silencing of global mRNA transcription. To test this idea, we expressed Impα2 in pole cells of embryos lacking maternal Pgc (*pgc impα2-nos3´UTR* embryos), and found that the frequency of *ftz* expression was drastically increased (pe = 51.4%, n = 74) (Fig 4F and 4G), compared to those of *impα2-nos3´UTR* embryos (P < 0.01) and the embryos lacking Pgc (*pgc* embryos) (pe = 34.9%, n = 109, P < 0.05) (Fig 4D, 4E and 4G). A similar situation was observed in embryos lacking both Pgc and Nos activities (*pgc nos* embryos) (Fig 5F and 5G). The percentage of embryos expressing *ftz* in pole cells was 82.8% (n = 209) (Fig 5H), an increase relative to 35.8% in *pgc* embryos (n = 203, P < 0.01) (Fig 5B, 5C and 5H), and 7.2% in *nos* embryos (n = 69, P < 0.01) (Fig 5D, 5E and 5H). Furthermore, ectopic *ftz* expression in *pgc nos* pole cells was suppressed by injecting double-stranded RNA (dsRNA) against *impα2* (Table 1). Therefore, we conclude that ectopic expression of *ftz* in pole cells is cooperatively repressed by Nos-dependent suppression of Impα2 production and Pgc.

**Table 1:** Ectopic *ftz* expression in *pgc nos* pole cells is suppressed by *impα2* knockdown.

Injected materials	No. of pole cells examined	No. of *ftz*-positive pole cells (%)		Significance
DW	55	14	(25.5)	
*impα2* dsRNA	45	3	(6.7)	*P* < 0.02

Embryos derived from *pgc/Df; nos/nos* females mated with *y w* males were injected with distilled water (DW) or dsRNA against *impα2* RNA at the cleavage stage. The injected embryos were allowed to develop until stage 4–6, and were stained for *ftz* mRNA. Six and five embryos injected with DW and dsRNA were examined, respectively. Significance was calculated using Fisher’s exact test.

In addition to *ftz* expression, *eve* was expressed ectopically in pole cells of *pgc impα2-nos3´UTR* embryos (S3 Fig). Ectopic *eve* mRNA and its protein expression were significantly higher in *pgc impα2-nos3´UTR* pole cells than *pgc* or *impα2-nos3´UTR* pole cells (S3 Fig). We next examined expression of the sex-determination gene *Sxl* in early pole cells, because *Sxl* is also repressed by *nos* in both male and female pole cells [15]. In males, *Sxl* mRNA expression was rarely detectable in pole cells of *nos*, *impα2-nos3´UTR*, *pgc*, and *pgc impα2-nos3´UTR* embryos (*P* > 0.1, vs. *y w*) (S4 Fig). By contrast, in females, the percentage of embryos expressing *Sxl* mRNA in pole cells was significantly higher in *pgc impα2-nos3´UTR* embryos than in *impα2-nos3´UTR*, and *pgc* embryos (S4 Fig). These results indicate that *eve* and *Sxl*, like *ftz*, are cooperatively repressed in pole cells by Impα2 depletion and Pgc-dependent transcriptional silencing. Because there is no evidence for the involvement of Ftz-F1 in *eve* and *Sxl* expression, it is likely that Impα2 mediates nuclear import of other transcriptional activator(s) for *eve* and/or *Sxl* in pole cells.

### Mis-expression of Impα2, unlike *nos* mutation, does not cause premature mitosis, apoptosis, or mis-migration of pole cells

**Fig 6:**
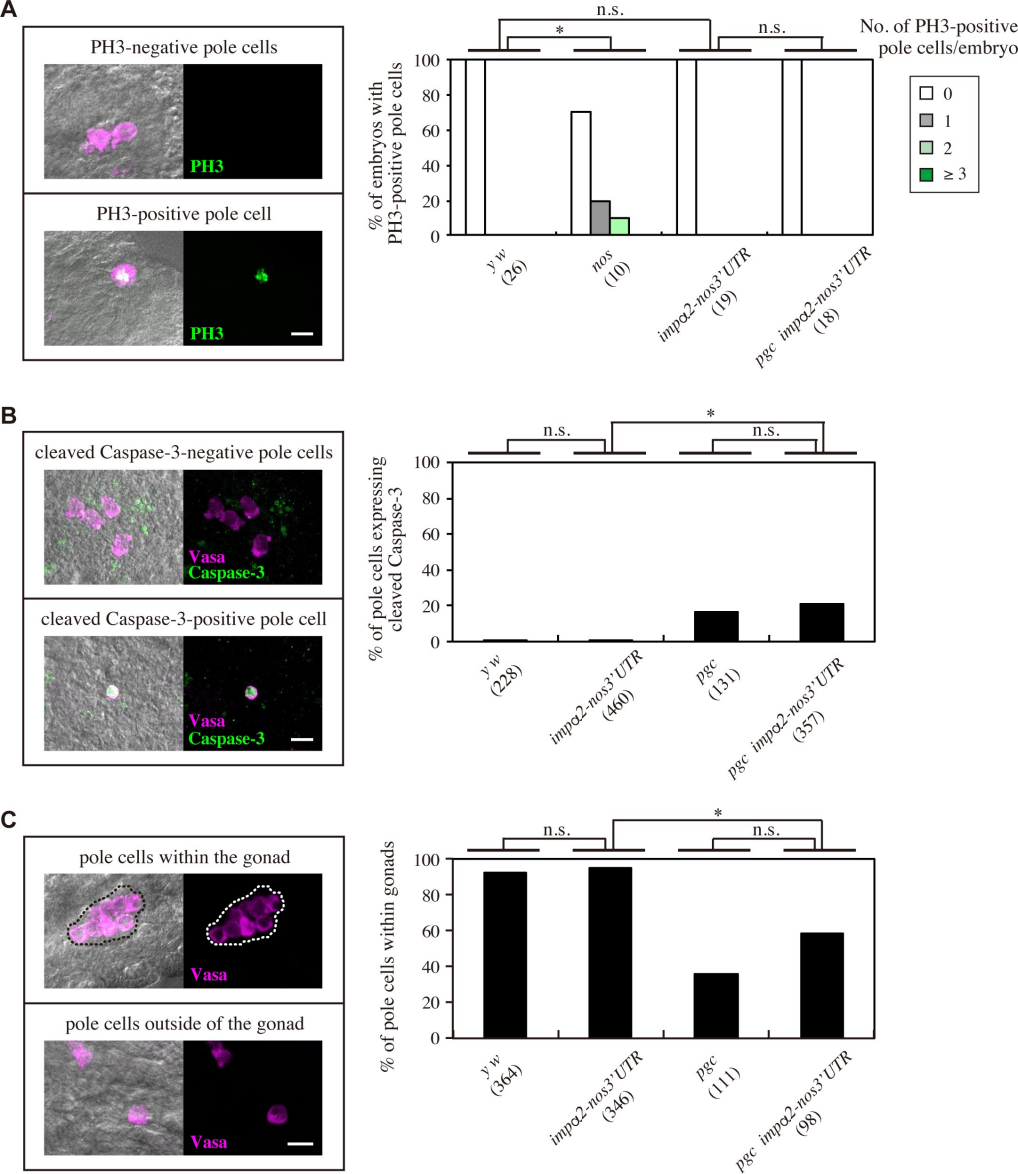
Mis-expression of Impα2 in pole cells has no significant effects on mitosis, apoptosis, or migration of pole cells. (**A**) Expression of a mitotic marker PH3 was examined in pole cells of embryos derived from *y w*, *nos*, *impα2-nos3’UTR*, and *pgc impα2-nos3’UTR* females mated with *y w* males. Stage-7–9 embryos were double-stained with anti-PH3 (green) and anti-Vasa (a marker for pole cells: magenta) antibodies. Left: representative images of pole cells negative (top) and positive (bottom) for PH3 in stage-8 embryos. DIC images merged with PH3- and Vasa-signals are also shown. Right: percentages of embryos carrying 0 (white), 1 (gray), 2 (pale green), or ≥3 (green) pole cells with PH3 signal are shown. The numbers of embryos examined are shown in parentheses. Significance was calculated using Fisher’s exact test (*: P < 0.05, n.s.: P > 0.5). (**B**) Expression of an apoptotic marker, cleaved Caspase-3, was examined in pole cells of embryos derived from *y w*, *impα2-nos3’UTR*, *pgc/pgc* (*pgc*), and *pgc impα2-nos3’UTR* females mated with *y w* males. Stage-10–16 embryos were double-stained with anti–cleaved Caspase-3 (green) and anti-Vasa (magenta) antibodies. Left: representative images of pole cells negative (top) and positive (bottom) for cleaved Caspase-3 in stage-12 embryos. DIC images merged with cleaved Caspase-3- and Vasa-signals are also shown. Right: percentages of pole cells expressing cleaved Caspase-3 are shown. The numbers of pole cells examined are shown in parentheses. For each genotype, 10–26 embryos were examined. Significance was calculated using Fisher’s exact test (*: P < 0.01, n.s.: P > 0.1). (**C**) Stage-14–16 embryos derived from *y w*, *impα2-nos3’UTR*, *pgc/Df* (*pgc*), and *pgc impα2-nos3’UTR* females mated with *y w* males were stained with anti-Vasa antibody (magenta). Left: representative images of pole cells within (top) and outside of (bottom) gonads in stage-14 embryos. DIC images merged with Vasa signal are also shown. Dotted line shows contour of embryonic gonads. Right: percentages of pole cells incorporated in the embryonic gonads. Total numbers of pole cells examined are shown in parentheses. For each genotype, 10–20 embryos were examined. Significance was calculated using Fisher’s exact test (*: P < 0.01, n.s.: P > 0.1). Scale bars, 10 μm (**A–C**).

Nos is required in pole cells for mitotic quiescence, repression of apoptosis, and proper migration to embryonic gonads [19, 43–45]. Hence, we asked whether mis-expression of Impα2 causes defects in these processes. First, using an antibody against a phosphorylated form of histone H3 (PH3), a marker of mitosis [46], we investigated whether pole cells enter mitosis in stage 7–9 embryos. Premature mitosis was detected in pole cells of *nos* embryos, as described previously [43], but never in pole cells of *impα2-nos3´UTR* or *pgc impα2-nos3´UTR* embryos (Fig 6A). Second, using an antibody against cleaved Caspase-3, a marker of apoptosis, we investigated whether pole cells enter apoptosis in stage 10–16 embryos. Pole cells never expressed the apoptotic marker in *impα2-nos3´UTR* embryos, whereas in *pgc impα2-nos3´UTR* embryos, 20.4% of pole cells expressed the apoptotic marker (Fig 6B). The latter was statistically indistinguishable from *pgc* pole cells (Fig 6B), which has been reported to enter apoptosis [47]. These data indicate that mis-expression of Impα2 does not affect apoptosis of pole cells even in the absence of *pgc* function. Last, we investigated whether mis-expression of Impα2 affects pole cell migration. The ability of pole cells to migrate properly into the embryonic gonads was never impaired in *impα2-nos3´UTR* embryos (Fig 6C), and the percentage of pole cells entering the gonads in *pgc impα2-nos3´UTR* embryos was statistically indistinguishable from that of *pgc* pole cells (Fig 6C), which has been reported to exhibit migration defect [47]. These observations indicate that mis-expression of Impα2 does not induce premature mitosis, apoptosis, or mis-migration of pole cells. This can be partly explained by the facts that *Cyclin B* and *hid* mRNAs are the targets for Nos-dependent translational repression regulating mitosis and apoptosis in pole cells, respectively [27, 43].

**Fig 7:**
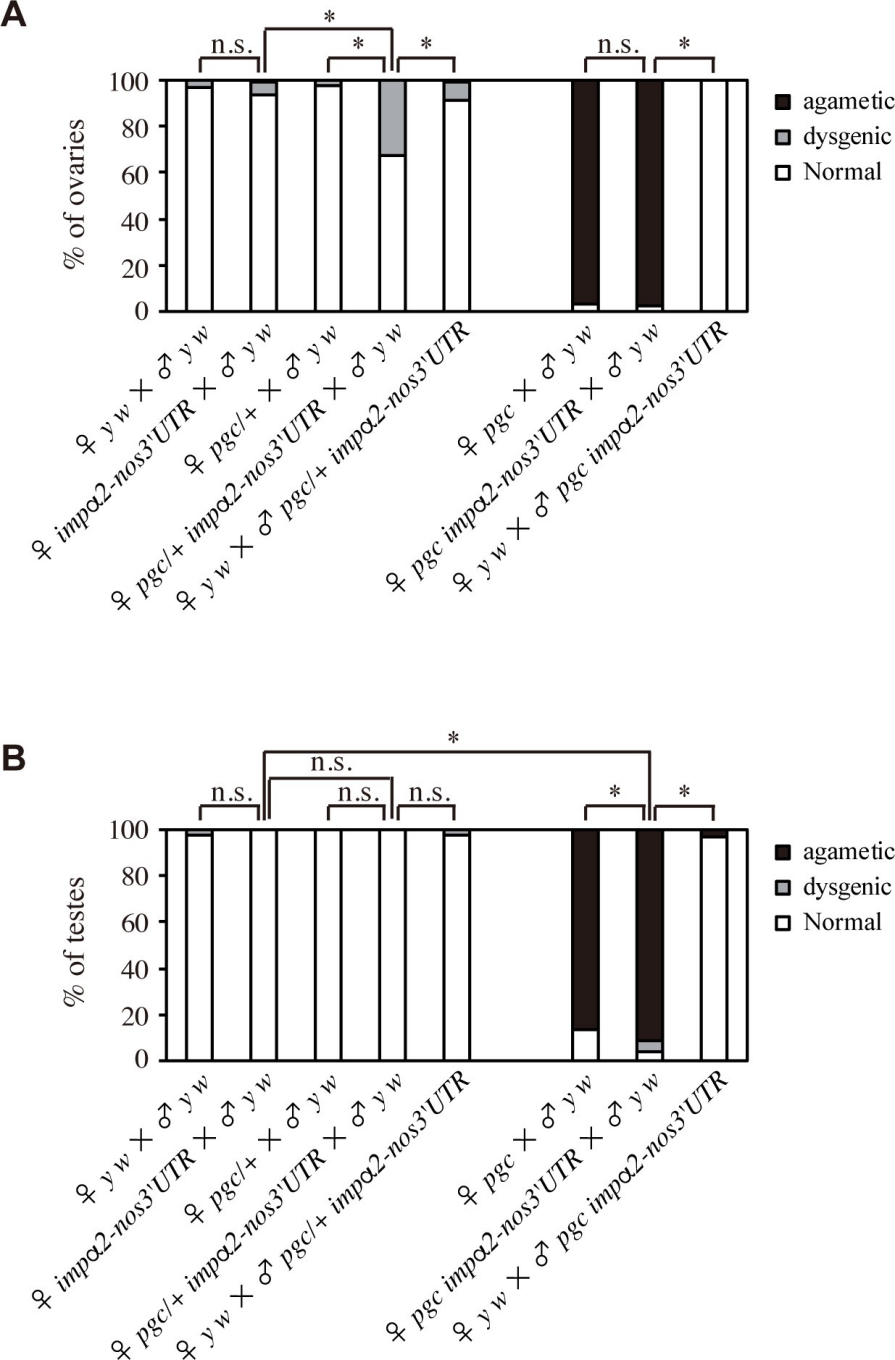
Mis-expression of Impα2 in pole cells affects gametogenesis. (**A** and **B**) Ovaries (**A**) and testes (**B**) of adults at 2–8 days after eclosion were examined. Flies were derived from *y w*, *impα2-nos3´UTR*, *pgc/+*, *pgc/+ impα2-nos3´UTR*, *pgc*, and *pgc impα2-nos3´UTR* females mated with *y w* males, or from *y w* females mated with *pgc/+ impα2-nos3´UTR* or *pgc impα2-nos3´UTR* males. Percentages of ovaries (**A**) and testes (**B**) showing normal (white), dysgenic (gray), and agametic (black) phenotypes (see S5 Fig) are shown. For each genotype, 88–190 ovaries and 68–252 testes were examined. Significance was calculated using Fisher’s exact test (*: P < 0.05, n.s.: P > 0.1).

During the course of the experiments described above, we happened to observe that *impα2-nos3´UTR* interacts genetically with the *pgc* mutation to cause dysgenic gametogenesis (Fig 7). Because almost all of the ovaries in females derived from *pgc* mothers mated with *y w* males were agametic, as reported previously [17], we examined the effect of *impα2-nos3´UTR* in *pgc/+* background (Fig 7A). The percentage of dysgenic ovaries in *pgc/+ impα2-nos3´UTR* females derived from *pgc*/+ *impα2-nos3´UTR* mothers mated with *y w* males was significantly higher than those in *pgc/+* and *impα2-nos3´UTR* females (Fig 7A). In the dysgenic ovaries, almost all of the egg chambers fail to complete the vitellogenic stage, and consequently only a few mature oocytes were present (S5 Fig). Furthermore, the percentages of dysgenic and agametic testes in *pgc impα2-nos3´UTR* males derived from *pgc impα2-nos3´UTR* mothers mated with *y w* males were higher than those in *pgc* and *impα2-nos3´UTR* males (Fig 7B). In these testes, the abundance of Vasa-positive germline cells was reduced (dysgenic) or absent (agametic) (S5 Fig). Because dysgenic and agametic gonads were barely detectable in females and males derived from reciprocal crosses (Fig 7), our data suggest that mis-expression of Impα2 from maternal transcript, concomitant with maternal *pgc* depletion in pole cells, causes defects in gametogenesis. However, we cannot test whether concomitant depletion of maternal Nos and Pgc causes a similar phenotype because *nos* pole cells degenerate before adulthood, even when apoptosis in these cells is genetically repressed [19].

### Mechanism of repression of somatic gene expression in pole cells by Nos and Pgc

Expression of Importin-α subtypes is spatio-temporally regulated in the soma during development in multiple animal species, including *Drosophila*, and they control nuclear transport of unique karyophilic proteins to activate different sets of somatic genes [30, 48–54]. *Drosophila* genome contains three Importin-α family genes: *impα1*, *2*, and *3* [28, 49, 55]. *impα1/Kap-α1/CG8548* mRNA is not detectable in pole cells during early embryogenesis [56, 57], and its protein product is ubiquitously expressed at a very low level throughout embryogenesis [48]. By contrast, maternal *impα3/Kap-α3/CG9423* mRNA is detectable in germ plasm during pole cell formation [58, 59], and production of Impα3 protein is upregulated during the blastoderm stage [55, 58] (S6 Fig). Because Impα3 production was independent of maternal *nos* activity (S6 Fig), it is likely that Nos-dependent repression of Impα2 production is solely responsible for suppression of somatic gene expression in pole cells. By contrast, pole cells become transcriptionally active during gastrulation [60–64], when Impα2 is undetectable in these pole cells [28]. Thus, the onset of zygotic transcription in pole cells may require Impα3-dependent nuclear import of transcription factors, in addition to the disappearance of Pgc and the alteration in chromatin-based regulation [10, 17]. After gastrulation, maternal *impα2* mRNA is rapidly degraded in pole cells, and neither *impα2* mRNA nor protein is detectable in the germline before adulthood [28]. This suggests that maternal *impα2* is dispensable for germline development, and that maternal *impα2* mRNA partitioned into early pole cells must be silenced by Nos and Pum in order to suppress mis-expression of somatic genes.

We found that depletion of maternal Nos activities caused mis-expression of *ftz* in pole cells. Although *ftz* expression was barely observed in pole cells lacking only maternal Nos, it was partially derepressed in pole cells in the absence of Pgc alone (Figs 4G and 5H), probably because a trace amount of Ftz-F1 enters pole cell nuclei even in the absence of the *impα2* translation. Therefore, we propose that a subset of somatic genes, including *ftz* and *eve*, are repressed in pole cells by two distinct mechanisms: Nos-dependent repression of nuclear import of transcriptional activators and Pgc-dependent silencing of mRNA transcription. Pgc inhibits P-TEFb-dependent phosphorylation of Ser2 residues in the heptad repeat of the C-terminal domain (CTD) of RNA polymerase II, a modification that is critical for transcriptional elongation [17]; thus, mRNA transcription in pole cells is globally suppressed by Pgc. By contrast, Nos inhibits transcription of particular genes by repressing Impα2-dependent nuclear import of the corresponding transcriptional activators.

Nos is evolutionarily conserved and expressed in the germline progenitors of various animal species [18]. In *C*. *elegans*, *nos-1* and *-2* are essential for rapid turnover of maternal *lin-15B* mRNA, which encodes a transcription factor that would otherwise cause inappropriate transcriptional activation in primordial germ cells [65]. In the germline progenitors of *Xenopus* embryos, Nos-1, along with Pum, destabilizes maternal *VegT* mRNA and represses its translation to inhibit somatic (endodermal) gene expression, which is activated by VegT protein [16]. Furthermore, in the germline progenitors (small micromeres) of sea urchin embryos, Nos silences maternal mRNA encoding a deadenylase, CNOT6, to stabilize other maternal mRNAs inherited into small micromeres [66]. Here, we demonstrate that Nos inhibits translation of maternal *impα2* mRNA in pole cells in order to suppress nuclear import of a transcriptional activator for somatic gene expression. Based on these observations, we propose that Nos silences maternal transcripts that are inherited into germline progenitors but deter the proper germline development. In addition to Nos-dependent silencing of maternal transcripts, transient suppression of RNA polymerase II elongation is observed during germline development of a wide range of animals, including *Drosophila*, *C*. *elegans*, *Xenopus*, and an ascidian, *Halocynthia roretzi* [17, 67–69]. Therefore, we propose that the ‘double-lock’ mechanism achieved by Nos and global suppression of RNA polymerase II activity plays an evolutionarily widespread role in germline development.

## Materials and methods

### *Drosophila* stocks

*y w* was used as a normal strain. *nos*^BN^/*nos*^BN^ [35, 70] or *nos*^*BN*^/*nos*^BN^ *Df(3L)H99* [19] were designated as *nos/nos*. *nos*^BN^/TM3 or *nos*^BN^/TM2 were designated as *nos/+*. *In(3R)Msc*/*T(1;3)FC8* [23, 71], *pgc*^Δ1^/*pgc*^Δ1^, *pgc*^Δ1^/*Df(2R)X58-7*, and *pgc*^Δ1^/*CyO* [17] are referred to as *pum/pum*, *pgc*/*pgc*, *pgc/Df*, and *pgc*/*+*, respectively. *nos*^BN^ and *In(3R)Msc*/*T(1;3)FC8* flies were gifts from R. Lehmann. *nos-gal4VP16* (*nos-gal4*) (a gift from R. Lehmann) [64] was used as a germline-specific driver. *y*^*1*^ *M{vas-int*.Dm}ZH-2A w*; M{3×P3-RFP.*attP}ZH-58A* (Bloomington Drosophila Stock Center, Stock No. 24484) was used as *y vas-φ-zh2A w*; *ZH-attP-58A* [72].

### Construction of *impα2-nos3´UTR*, *UASp-impα2 WT*, and *UASp-impα2 ΔNRE* transgenes and germline transformation

#### impα2-nos3´UTR

The full-length *impα2* coding region was amplified from an *impα2* cDNA clone *K9* (a gift from B. M. Mechler) [28], which contains 212 bp of 5´ UTR, 1569 bp of protein-coding region, and the entire 624-bp 3´ UTR of *impα2* [nucleotides (ntd) 79–2483 of GenBank accession no. BT003258] using PCR primers 5´-CATATGAGTAAGGCGGATTCTAA-3´ (*impα2*−*5´*) and 5´-CATATGTTAGAACGTGTAGCCACC-3´ (*impα2*−*3´*); the underlined sequences are *Nde*I sites. The amplified fragment was subcloned into pBS-Pnos-nos3´UT [43], a derivative of pBS-KS Pnos and pBS-KSnos3´UT (gifts from E. Gavis), which contains 750 bp of *nos* promoter, 263 bp of the *nos* 5´ UTR, 880 bp of the *nos* 3´ UTR, and 75 bp of the 3´ flanking region of the *nos* gene. The amplified *impα2* cDNA fragment (*Nde*I–*Nde*I) was inserted into a unique *Nde*I site (CATATG) in pBS-Pnos-nos3´UT; the resultant chimeric gene contains an AUG only at the position immediately downstream of the *nos* 5´ UTR. Then, a *Kpn*I–*Not*I fragment containing the entire *Pnos-impα2-nos3´UTR* chimeric gene was subcloned into pCaSpeR4 [73] for transformation.

#### *UASp-impα2 WT* and *UASp-impα2 ΔNRE*

The 3´ fragment of *impα2* cDNA containing 54 bp of protein-coding region and the entire 624-bp 3´ UTR region was amplified from *impα2* cDNA clone *K9* using the following primers: 5´-CTCGAGTTCAATGCCACCCAGCCCAAGGCTCCCGAAGGTGGCTACACGTTCtaaTCGCCCACCCCACACATTCC-3´ (*XhoI-oho-FW1*, ntd 1806–1879 of GenBank accession no. BT003258) and 5´- 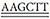
TTTTTTTTTTTTTTTTTTTTTTTAATCATTCA*TCATTATTTATTG*TTTGAATATAAACATGCGATTCGGG-3´ (*HindIII-pA-NRE(Δ)-RV1*, complementary to ntd 2426–2483 of GenBank accession no. BT003258). (In the sequence given in the previous sentence, the stop codon is in lowercase, the unique *Xho*I site in the coding region is underlined, and the *Hind*III site is double-underlined; the positions of the deleted NRE sequences are marked by asterisks.) The *Xho*I–*Hind*III fragment of the resultant amplicon was subcloned between the *Xho*I and *Hind*III sites of clone *K9* to replace a 677-bp 3´ fragment of *impα2* cDNA. The resultant clone, *K9ΔNRE*, contains full-length *impα2* cDNA lacking the NRE sequence in its 3´ UTR. A triple Myc tag sequence was inserted immediately before the stop codon of the *impα2* cDNA fragment (clone *K9*) or *impα2ΔNRE* fragment (clone *K9ΔNRE*) by inverse PCR (iPCR) using the KOD Plus Mutagenesis Kit (Toyobo) with the following primers: 5´-**GATTAATTTTTGTTCCAAGTCTTCCTCGGAGATTAGCTTTTGTTC**GAACGTGTAGCCACCTTCGGGAGCC-3´ (*Myc-imp -RV1*) and 5´-**TCAGAAGAAGACTTGGAACAAAAGTTGATTTCTGAAGAAGATTTG**TAATCGCCCACCCCACACATTCCAAAC-3´ (*Myc-imp -FW1*). The Myc tag sequence is in bold. The resultant Myc-tagged full-length cDNA fragments were amplified using the following primers: 5´-GG
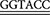
AGCGTGTTAGCACGCTCGAC-3´ (*KpnI-oho-5´-FW4*) and 5´-ATTT
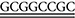
AATCATTCAAACAATTCATTATTTATTGAC-3´ (*NotI-oho-NRE(W)-RV4*) or 5´-ATTT
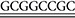
AATCATTCA*TCATTATTTATTG*TTTGAATA-3´ (*NotI-oho-NRE(Δ)-RV4*). (The *Kpn*I and *Not*I sites are underlined and double-underlined, respectively; the positions of the deleted NRE sequences are marked by asterisks.) The resultant amplicon was subcloned between the *Kpn*I and *NotI* sites of pUASp-K10 attB [74].

The nucleotide sequences of the above constructs were confirmed by sequencing, and then the constructs were transformed into flies. To establish *impα2-nos3´UTR* flies, germline transformation was performed as described previously [75] using *y w* embryos as recipients. Two independent *w*^+^ transformants for each transgene were mated with *y w* females to establish homozygous stocks. Data shown in figures were obtained from one of the two independent transformant lines, as we found no significant difference between the two lines. To establish *UASp-impα2 WT* and *UASp-impα2 ΔNRE* flies, germline transformation was performed using embryos derived from *y vas-φ-zh2A w*; *ZH-attP-58A* females [72], and a single transformant line was established for each transgene, as described previously [76].

### Staging of embryos

Developmental stages of *Drosophila* embryos were determined according to Campos-Ortega and Hartenstein [77]. In this study, stage-4 embryos that had finished the 13th somatic nuclear division and retained round nuclei before cellularization were referred to as "late stage-4 embryos".

### Immunostaining

Antibody staining of embryos was performed as described previously [43]. For anti-Impα2 staining, embryos were fixed in 2 ml of 1:1 mixture of heptane and fixative I [3.7% formalin in PBS (130 mM NaCl, 7 mM Na_2_HPO_4_, 3 mM NaH_2_PO_4_)] for 10 min with vigorous shaking. Two different antibodies were used, anti-Impα2 23aa and anti-Impα2 2/3 (gifts from B. M. Mechler), which were raised against the 23-amino acid residues of the C-terminal region and two-thirds of Impα2 protein, respectively [28, 48]. For the experiments shown in Fig 1, rabbit anti-Impα2 23aa antibody (1:50 dilution) and Alexa Fluor 488-conjugated anti-rabbit IgG antibody (1:200 dilution, Molecular Probes) were used. For the experiments shown in S1 Fig, rabbit anti-Impα2 2/3 antibody (1:40 dilution) and biotinylated anti-rabbit IgG antibody (1:200 dilution, Vector Lab.) were used. The signal was amplified using Vectastain ABC-AP kit (Vector Lab.), and then detected with 5-bromo-4-chloro-indolyl phosphate (BCIP)/nitroblue tetrazolium (NBT) (Boehringer Mannheim). Embryos were dehydrated in graded alcohol and mounted in Eukitt (O. Kindler). We observed no significant difference in the results obtained using these two antibodies, except that anti-Impα2 23aa antibody often caused a non-specific signal on the embryo surface.

For double-staining with anti-Ftz-F1 antibody and propidium iodide (Fig 3), embryos were fixed in 2 ml of 1:1 mixture of heptane and fixative II (4% paraformaldehyde in PBS) for 5 min with vigorous shaking. Rabbit anti-Ftz-F1 antibody (1:500 dilution, a gift from H. Ueda) and Alexa Fluor 488-conjugated anti-rabbit IgG antibody (1:500 dilution, Molecular Probes) were used. The embryos were treated with RNase, and then stained with propidium iodide (Sigma), as described previously [43]. For double-staining with anti-Ftz-F1 antibody and DAPI, the embryos were treated with DAPI (1 μg/ml, Sigma) for 10 min, after anti-Ftz-F1 staining.

For anti-Eve staining, embryos were fixed in 2 ml of 1:1 mixture of heptane and fixative II for 5 min with vigorous shaking. Guinea pig anti-Eve antibody 634 [1:200 dilution, Asian Distribution Center for Segmentation Antibodies at National Institute of Genetics (NIG), Japan] [78] and Cy3-conjugated anti-guinea pig IgG antibody (1:500 dilution, Jackson ImmunoResearch) were used.

For the experiments shown in Fig 2, embryos were fixed in 2 ml of 1:1 mixture of heptane and fixative I for 20 min. Mouse anti-Myc antibody 9E10 [1:100 dilution, Developmental Studies Hybridoma Bank (DSHB) at the University of Iowa] and HRP (horse-radish peroxidase)-conjugated anti-mouse IgG antibody (1:500 dilution, Bio-Rad) were used. The signal was enhanced using the TSA-Biotin System and Streptavidin-FITC (PerkinElmer Life Sciences, Inc.).

For staining with antibodies against Vasa, PH3, cleaved Caspase-3, and Impα3, embryos were fixed in 2 ml of 1:1 mixture of heptane and fixative II for 20 min. The following antibodies were used: chick anti-Vasa antibody (1:500 dilution, lab stock), rabbit anti-PH3 antibody (1:200 dilution, Upstate Biotechnology), rabbit anti–Caspase-3 antibody ab13847 (lot no. 593692, 1:1000 dilution, Abcam), and mouse anti–dKap-α3 antibody 5E3 (1:500 dilution, a gift from C. S. Parker). Signal was detected using Cy3-conjugated anti–chick IgY antibody (1:500 dilution, Jackson ImmunoResearch), Alexa Fluor 488–conjugated anti–rabbit IgG antibody A-11034 (1:500 dilution, Molecular Probes), or Alexa Fluor 488–conjugated anti–mouse IgG antibody A-11029 (1:500 dilution, Molecular Probes), as appropriate.

Antibody staining of ovaries and testes was performed as previously described for the ovary [79]. Chick anti-Vasa antibody (1:500 dilution, lab stock) and Alexa Fluor 488–conjugated anti–chick IgY antibody A-11039 (1:500 dilution, Molecular Probes) were used.

All embryos, ovaries, and testes stained with fluorochrome-conjugated secondaries were mounted in Vectashield (Vector Laboratories) or ProLong Diamond (Molecular Probes). Z-stack confocal images were taken from each embryo using a Zeiss LSM 5 Pascal (Zeiss), Zeiss LSM 510 Meta (Zeiss), Leica TCS-NT (Leica), or Leica TCS-SP8 (Leica) confocal microscope. Optical slices were analyzed using Zeiss LSM 5 Image Browser (Zeiss), ImageJ, or Fiji software. In Figs 2F, 3J, 6A and S1, the numbers of signal-positive pole cells located from the top to median plane of embryos were counted in confocal serial images.

### *In situ* hybridization

Digoxigenin (DIG)-labeled RNA probes were synthesized with SP6, T7, or T3 RNA polymerase in the presence of DIG-labeled uridine triphosphate (UTP) (Boehringer-Mannheim), using full-length *impα2* cDNA clone *K9*, full-length 1817-bp *ftz* cDNA (a gift from H. Ueda), a 985-bp *eve* cDNA fragment (ntd 231–1215 of GenBank accession no. BT029151), or an 848-bp *Sxl* cDNA fragment (ntd 572–1419 of GenBank accession no. NM167112) as the template. Whole-mount *in situ* hybridization of embryos was performed essentially according to the methods reported by Tautz and Pfeifle [80], with several modifications [81]. For staining with *impα2* probe, fixed embryos were treated at 23°C for 3 min with PBT (130 mM NaCl, 7 mM Na_2_HPO_4_, 3mM NaH_2_PO_4_, 0.1% Tween-20) containing 50 μg/ml Proteinase K, and the reaction was immediately stopped by treating twice for 30 sec each with PBT containing 2 mg/ml glycine. Hybridization was performed for 16 hr at 60°C in hybridization solution (50% formamide, 5 x SSC, 0.1% Tween 20, 0.05 mg/ml heparin, 0.1 mg/ml yeast tRNA) containing 0.6 μg/ml *impα2* RNA probe. Post-hybridization washing was performed six times (30 min each) at 60°C in a solution containing 50% formamide, 5× SSC, and 0.1% Tween-20. Embryos were incubated for 30 min with Fab fragments of anti-DIG antibody conjugated with HRP (600 U/l, Boehringer-Mannheim), then the signal was enhanced using the TSA-Biotin System (PerkinElmer Life Sciences, Inc.) and Streptavidin-Cy3 conjugate (1:2000 dilution, Jackson ImmunoResearch). For staining with *ftz*, *eve* or *Sxl* probe, the fixed embryos were treated at room temperature for 15 min with PBT containing 7 μg/ml Proteinase K, and then the reaction was stopped as described above. Hybridization was performed for 16 hr at 56°C in hybridization solution containing 0.5 μg/ml of *ftz*, *eve* or *Sxl* RNA probe. The embryos were washed five times (30 min each) at 56°C in hybridization solution, and then rinsed in PBT containing 75%, 50%, and 25% hybridization solution for 5 min each, and in PBT five times for 5 min each. The embryos were incubated with HRP-conjugated anti-DIG antibody (300 U/l) for 16 hr at 4°C, and the signal was enhanced using TSA-Plus Fluorescein System (PerkinElmer Life Sciences, Inc.). Embryos were mounted in Vectashield (Vector Laboratories) or ProLong Diamond (Molecular Probes). Z-stack confocal images were taken from each embryo using a Zeiss LSM 5 Pascal (Zeiss), Leica TCS-NT (Leica), or Leica TCS-SP8 (Leica) confocal microscope. Optical slices were analyzed using Zeiss LSM 5 Image Browser (Zeiss), or Fiji software. In Figs 4G, 5H, S3 and S4, the numbers of signal-positive pole cells located from the top to median plane of embryos were counted in confocal serial images.

### Quantification of Impα2 and Impα3 signals and nuclear localization of Ftz-F1

Embryos from late stage 4 to stage 6 were stained with anti-Impα2 23aa antibody. Serial optical sections (1.3 μm thick, 4–5 sections per pole cell) were obtained using a confocal microscope (LSM 510 Meta, Zeiss). Embryos from late stage 4 to stage 5 were stained with anti-Impα3 antibody, and serial optical sections (1.0 μm thick, 6–8 sections per pole cell) were obtained using a TCS-SP8 confocal microscope (Leica). Fluorescence intensities from the area occupied by individual pole cells (judged by the outline of the cell in the DIC image) were determined in sections through the median plane of pole cells. Fluorescence intensities were measured in all pole cells located within 15 μm of the top section of confocal serial images. Average fluorescence intensities (intensity/pixel) were calculated.

Embryos from late stage 4 to stage 5 were double-stained with anti-Ftz-F1 antibody and propidium iodide or DAPI as described above. Under a confocal microscope (Zeiss LSM 510 Meta, Zeiss), serial optical sections (1.3 μm thick, 4–5 sections per pole cell) were obtained. We examined all pole cells located within 18.2 μm of the top section of confocal serial images. To quantify Ftz-F1 distribution in the nucleus of a single pole cell, fluorescence intensities from the area occupied by the nucleus were determined for each section using ImageJ, and then summed. The nuclear area was judged as the propidium iodide- or DAPI-positive area. To quantify Ftz-F1 distribution in the cytosol of pole cells, we measured fluorescence intensity from the whole area of a pole cell (judged by the outline of the cell in the DIC image), and then fluorescence intensity of the cytosolic area was calculated by subtracting the nuclear intensity from the whole-cell intensity. Average fluorescence intensities (intensity/pixel) were calculated for both nuclear and cytoplasmic areas, and the ratio of nuclear to cytoplasmic intensity was calculated.

### Injection of double-stranded RNA (dsRNA) against *impα2* mRNA

Template DNA was amplified from *impa2* cDNA clone *K9* by PCR using forward primer 5´-**GCGCGAATTAACCCTCACTAAAGGG**CTCCCGAACAGATCGTCG-3´ (ntd 1483–1500 of GenBank accession no. BT003258) and reverse primer 5´- **GCGCGAATTAACCCTCACTAAAGGG**AATCATTCAAACAATTCATTATTTATTGACAACTTTG-3´ (complementary to ntd 2447–2483 of GenBank accession no. BT003258), both of which contain the promoter sequences for T3 RNA polymerase (shown in bold) at their 5´-ends. dsRNA was transcribed *in vitro* from the amplified DNA with T3 RNA polymerase (MEGAscript T3 kit, Ambion). dsRNA (0.1 nl of a 1.7 μg/μl solution) was injected into the posterior pole of *pgc nos* embryos at early stage 2. Because knockout of maternal *impα2* mRNA results in developmental arrest at early cleavage stage [82], we performed partial knockdown of *impα2* mRNA by precisely regulating the injection volume using a thin glass needle (hole diameter = 3 μm). Injected embryos were fixed in a 1:1 mixture of heptane and fixative II for 20 min, and the vitelline membrane was removed in PBS using a tungsten needle. Fixed embryos were processed for *in situ* hybridization with an antisense *ftz* RNA probe, as described above. The pole cells located within 30 μm of median section of confocal serial images were counted.

### Nos and Pum protein purification

Recombinant Nos and Pum proteins were expressed in KRX *E*. *coli* cells (Promega) as described previously [22] using the Nos expression plasmid pFN18K NosZC (aa 289–401) (a gift from A. C. Goldstrohm) and the Pum expression plasmid pFN18K Pum RNA-binding domain (aa 1091–1426) (a gift from A. C. Goldstrohm). For Nos expression, cells were cultured in 2×YT medium with 25 μg/ml kanamycin and 2 mM MgSO_4_ at 37°C to an OD_600_ of 0.7–0.9, and then protein expression was induced with 0.1% (w/v) rhamnose for 3 hr. For Pum expression, cells were cultured at 37°C in the same medium to an OD_600_ of 0.6, and then at 16°C to an OD_600_ of 0.7–0.9. Protein expression was induced with 0.1% rhamnose for 14–16 hr at 16°C. Nos and Pum proteins were purified essentially as described by Weidmann *et al*. [22], with the following modifications. Nos and Pum proteins with Halo tag were purified by incubating with Magne HaloTag beads (Promega) overnight at 4°C. Beads were washed three times with Wash Buffer (50 mM Tris-HCl pH 8.0, 2 mM MgCl_2_, 1 M NaCl, 1 mM DTT, 0.5% [v/v] NP-40), and three times with Elution Buffer (50 mM Tris-HCl pH 7.6, 150 mM NaCl, 1 mM DTT, 20% [v/v] glycerol). Then, the beads were resuspended in Elution Buffer containing AcTEV protease (Invitrogen) and incubated for 24 hr at 4°C to cleave Nos or Pum protein from the Magne HaloTag beads. The beads were then removed using a MagneSphere magnetic separation stand (Promega).

### Electrophoretic mobility shift assay (EMSA)

Synthetic Cy5-labeled *impα2* RNA fragment (IDT, Tokyo), shown in S2 Fig, were used in EMSA. RNA-binding reactions were performed in RNA-binding buffer (50 mM Tris-HCl pH 7.6, 150 mM NaCl, 2mM DTT, 2 μg/ml BSA, 0.01% [v/v] NP-40, 20% [v/v] glycerol). Target RNA (100 nM), purified Pum (1.2 μM), and Nos (1.2 μM) were incubated in RNA binding buffer for 3 hr at 4°C. Native polyacrylamide TBE mini-PROTEAN gel (5%, Bio-Rad) was pre-run for 2.5 hr at 50 V, and then 10 μl of each sample was loaded and the gel was run at 50 V for 2 hr 10 min at 4°C. A Typhoon FLA 7000 laser scanner (GE Healthcare) was used to image EMSA.

## Supporting information

S1 Fig Nos and Pum repress mis-expression of Impα2 in pole cells. Expression of Impα2 was examined in pole cells of embryos derived from *nos/+*, *nos/nos* (*nos*), *pum*^Msc^/*TM3* (*pum*^Msc^/+), *pum*^FC8^/*TM3* (*pum*^FC8^/+), and *pum*^Msc^/*pum*^FC8^ (*pum*) females. Embryos from late stage 4 to stage 6 were stained with anti-Impα2 2/3 antibodies [28]. Percentages of embryos containing 0 (white), 1–3 (pale orange), 4–6 (orange), and ≥7 (red) pole cells with Impα2 signal are shown. The numbers of embryos examined are shown in parentheses. Significance was calculated using Fisher’s exact test (*: P < 0.01).
(TIF)

S2 Fig Nucleotide sequences of RNAs used in EMSA. The nucleotide sequence of *impα2* RNA fragment containing wild-type (*WT*) or mutated (*mut*) NRE-like sequence, used in Fig 2H, is shown. The NRE-like sequence is boxed, and UGU is marked by blue letters. The substituted nucleotides in the *mut* RNA are marked by red. Nos-Pum SEQRS motifs [22] are shown above the nucleotide sequences.
(TIF)

S3 Fig Mis-expression of Impα2 results in ectopic eve expression in pole cells lacking Pgc. (**A**) Expression of *eve* mRNA was examined in pole cells of embryos from late stage 4 to stage 5. Embryos were derived from *y w* females with (*impα2-nos3’UTR*) or without two copies of *impα2-nos3’UTR* (*y w*), and *nos/nos* (*nos*), *pgc/Df* (*pgc*), and *pgc/pgc; impα2-nos3’UTR/impα2-nos3’UTR* (*pgc impα2-nos3’UTR*) females mated with *y w* males. Percentages of embryos carrying 0 (white), 1 (gray), 2–4 (pale green), or ≥5 (green) pole cells with *eve* mRNA signal are shown. The numbers of embryos examined are shown in parentheses. Significance was calculated using Fisher’s exact test (*: P < 0.05, n.s.: *P* > 0.5). (**B**) Expression of Eve protein was examined in pole cells of embryos from late stage 4 to stage 5. Embryos were derived from *y w*, *nos*, *impα2-nos3’UTR*, *pgc* and *pgc impα2-nos3’UTR* females mated with *y w* males, as described above. Percentages of embryos carrying 0 (white), 1–3 (pale orange), 4–6 (orange), or ≥7 (red) pole cells with Eve signal are shown. The numbers of embryos examined are shown in parentheses. Significance was calculated using Fisher’s exact test (*: P < 0.01, n.s.: *P* > 0.1).
(TIF)

S4 Fig Mis-expression of Impα2 results in ectopic expression of Sxl mRNA in pole cells lacking Pgc. (**A, B**) Expression of *Sxl* mRNA was examined in pole cells of female (**A**) and male (**B**) embryos at late stage 4 to stage 5. Embryos were derived from *y w*, *nos*, *impα2-nos3’UTR*, *pgc/Df* (*pgc*), and *pgc impα2-nos3’UTR* females mated with *y w* males. Sex of the embryos was judged by expression of *Sxl* mRNA in the soma, where strong expression of *Sxl* was observed in female, but not in male. Percentages of embryos carrying 0 (white), 1 (gray), 2–4 (pale green), or ≥5 (green) pole cells with *Sxl* mRNA signal are shown. The numbers of embryos examined are shown in parentheses. Significance was calculated using Fisher’s exact test (*: P < 0.05, n.s.: *P* > 0.1).
(TIF)

S5 Fig Phenotypes observed in adult gonads. (**A–F**) Representative images of normal (**A, D**), dysgenic (**B, E**), and agametic (**C, F**) ovaries. Ovaries of adults (3–5 days after eclosion) were stained for Vasa (a germline marker, green). Bright field images (**A–C**) and confocal images (**D–F)** are shown. In normal ovaries, oogenesis progressed properly, resulting in production of many mature oocytes (**A, D**). By contrast, in dysgenic ovaries, egg chambers were degenerated during vitellogenesis, and only a few mature oocytes formed (**B, E**). Agametic ovaries contain no germline cells (**C, F**). (**G–I**) Representative images of distal-tip regions of normal (**G**), dysgenic (**H**), and agametic (**I**) testes. Testes of adults (2–5 days after eclosion) were stained for Vasa (green). In normal testes, spermatogenesis progressed properly (**G**). By contrast, dysgenic (**H**) and agametic (**I**) testes contained few and no Vasa-positive germline cells, respectively. Scale bars, 500 μm (**C**), 200 μm (**F**), and 20 μm (**I**).
(TIF)

S6 Fig Depletion of Nos has no significant effect on Impα3 protein expression in pole cells. (**A**) Fluorescence intensities of Impα3 protein signals in pole cells of embryos derived from *y w* and *nos* females. Embryos from late stage 4 to stage 5 were stained with anti-Impα3 antibody, and fluorescence intensities of Impα3 signals were measured (see Materials and Methods). Mean values of fluorescence intensities (± SE) are shown. The numbers of pole cells measured are shown in parentheses. 12 and 10 embryos were examined for *y w* and *nos*, respectively. Significance was calculated using paired t-test (n.s.: *P* > 0.1). (**B, C**) Stage-5 embryos derived from *y w* (**A**) and *nos* (**B**) females were stained for Impα3 protein. In pole cells, as well as in somatic cells, Impα3 was mainly detected on the nuclear envelope and in the nuclei [58]. Arrows and arrowheads point to pole cells expressing Impα3 on the nuclear envelope, with or without signal in their nuclei, respectively. Scale bar, 10 μm.
(TIF)
