# Supplementary figures and images for: Maternal Nanos inhibits Importin-α2/Pendulin-dependent nuclear import to prevent somatic gene expression in the Drosophila germline
Source: PLoS Genet. 2019 May 15;15(5):e1008090. doi: 10.1371/journal.pgen.1008090 (PMC6519790; doi:10.1371/journal.pgen.1008090)

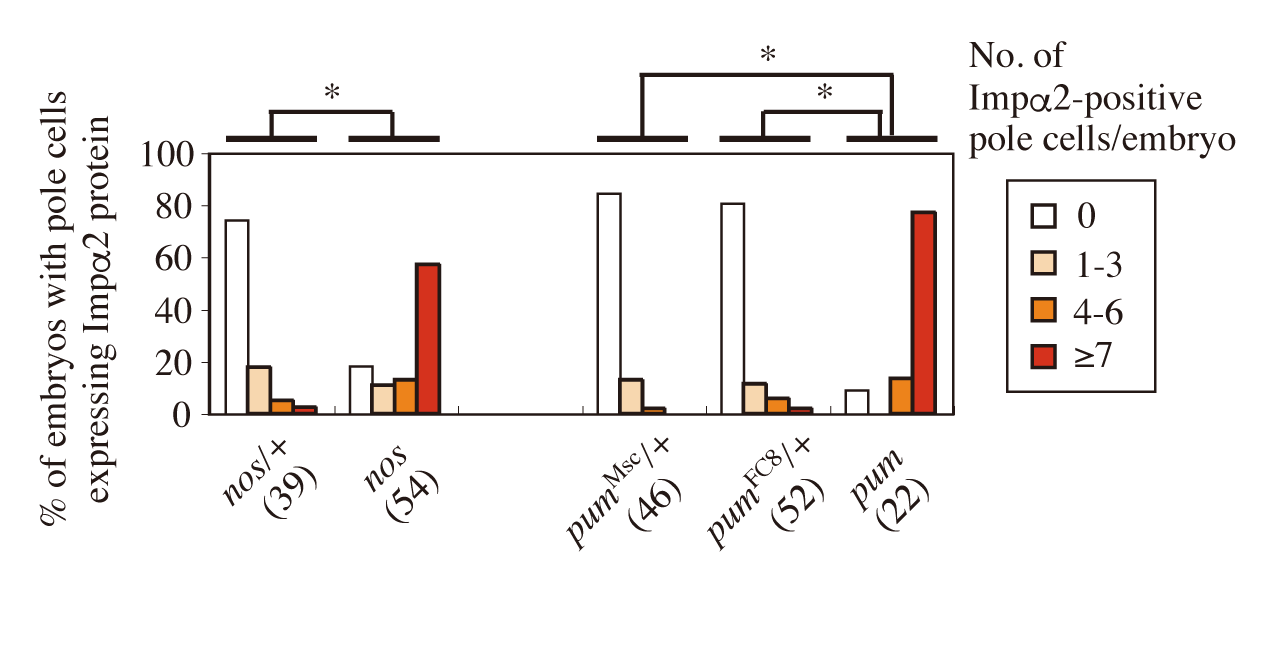

Supplement: S1 Fig — Expression of Impα2 was examined in pole cells of embryos derived from nos/+, nos/nos (nos), pumMsc/TM3 (pumMsc/+), pumFC8/TM3 (pumFC8/+), and pumMsc/pumFC8 (pum) females. Embryos from late stage 4 to stage 6 were stained with anti-Impα2 2/3 antibodies [28]. Percentages of embryos containing 0 (white), 1–3 (pale orange), 4–6 (orange), and ≥7 (red) pole cells with Impα2 signal are shown. The numbers of embryos examined are shown in parentheses. Significance was calculated using Fisher’s exact test (*: P < 0.01). (TIF) [file pgen.1008090.s001.tif]

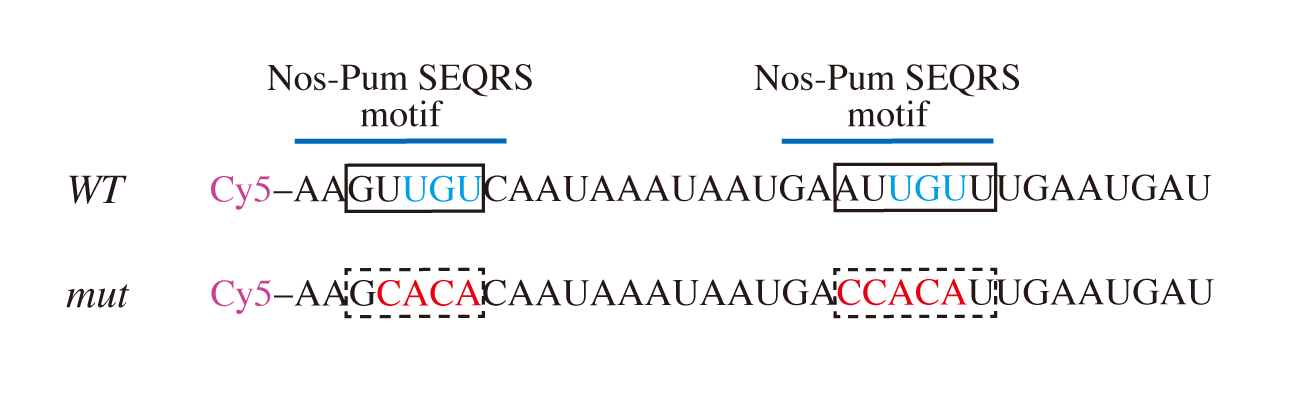

Supplement: S2 Fig — The nucleotide sequence of impα2 RNA fragment containing wild-type (WT) or mutated (mut) NRE-like sequence, used in Fig 2H, is shown. The NRE-like sequence is boxed, and UGU is marked by blue letters. The substituted nucleotides in the mut RNA are marked by red. Nos-Pum SEQRS motifs [22] are shown above the nucleotide sequences. (TIF) [file pgen.1008090.s002.tif]

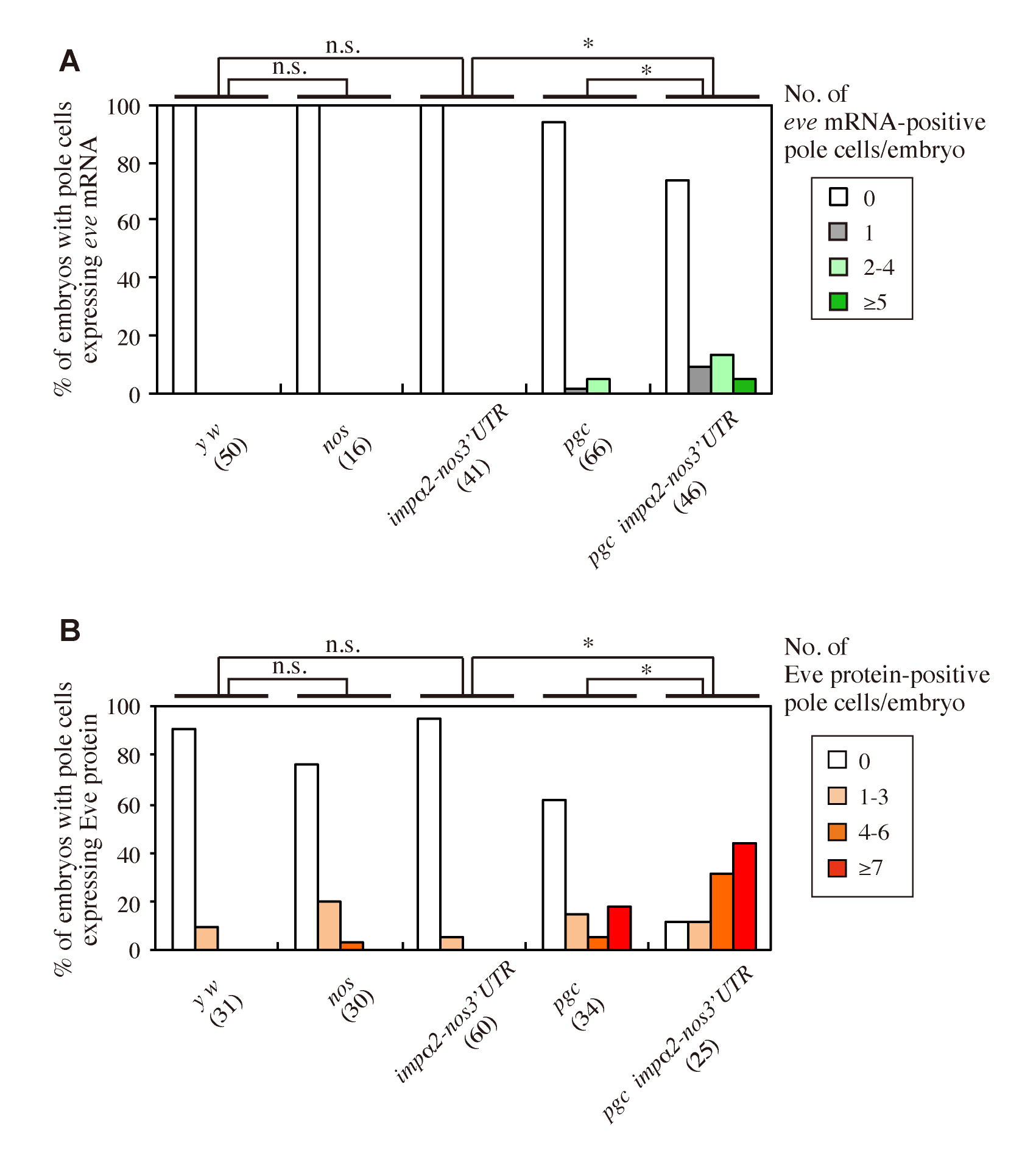

Supplement: S3 Fig — (A) Expression of eve mRNA was examined in pole cells of embryos from late stage 4 to stage 5. Embryos were derived from y w females with (impα2-nos3’UTR) or without two copies of impα2-nos3’UTR (y w), and nos/nos (nos), pgc/Df (pgc), and pgc/pgc; impα2-nos3’UTR/impα2-nos3’UTR (pgc impα2-nos3’UTR) females mated with y w males. Percentages of embryos carrying 0 (white), 1 (gray), 2–4 (pale green), or ≥5 (green) pole cells with eve mRNA signal are shown. The numbers of embryos examined are shown in parentheses. Significance was calculated using Fisher’s exact test (*: P < 0.05, n.s.: P > 0.5). (B) Expression of Eve protein was examined in pole cells of embryos from late stage 4 to stage 5. Embryos were derived from y w, nos, impα2-nos3’UTR, pgc and pgc impα2-nos3’UTR females mated with y w males, as described above. Percentages of embryos carrying 0 (white), 1–3 (pale orange), 4–6 (orange), or ≥7 (red) pole cells with Eve signal are shown. The numbers of embryos examined are shown in parentheses. Significance was calculated using Fisher’s exact test (*: P < 0.01, n.s.: P > 0.1). (TIF) [file pgen.1008090.s003.tif]

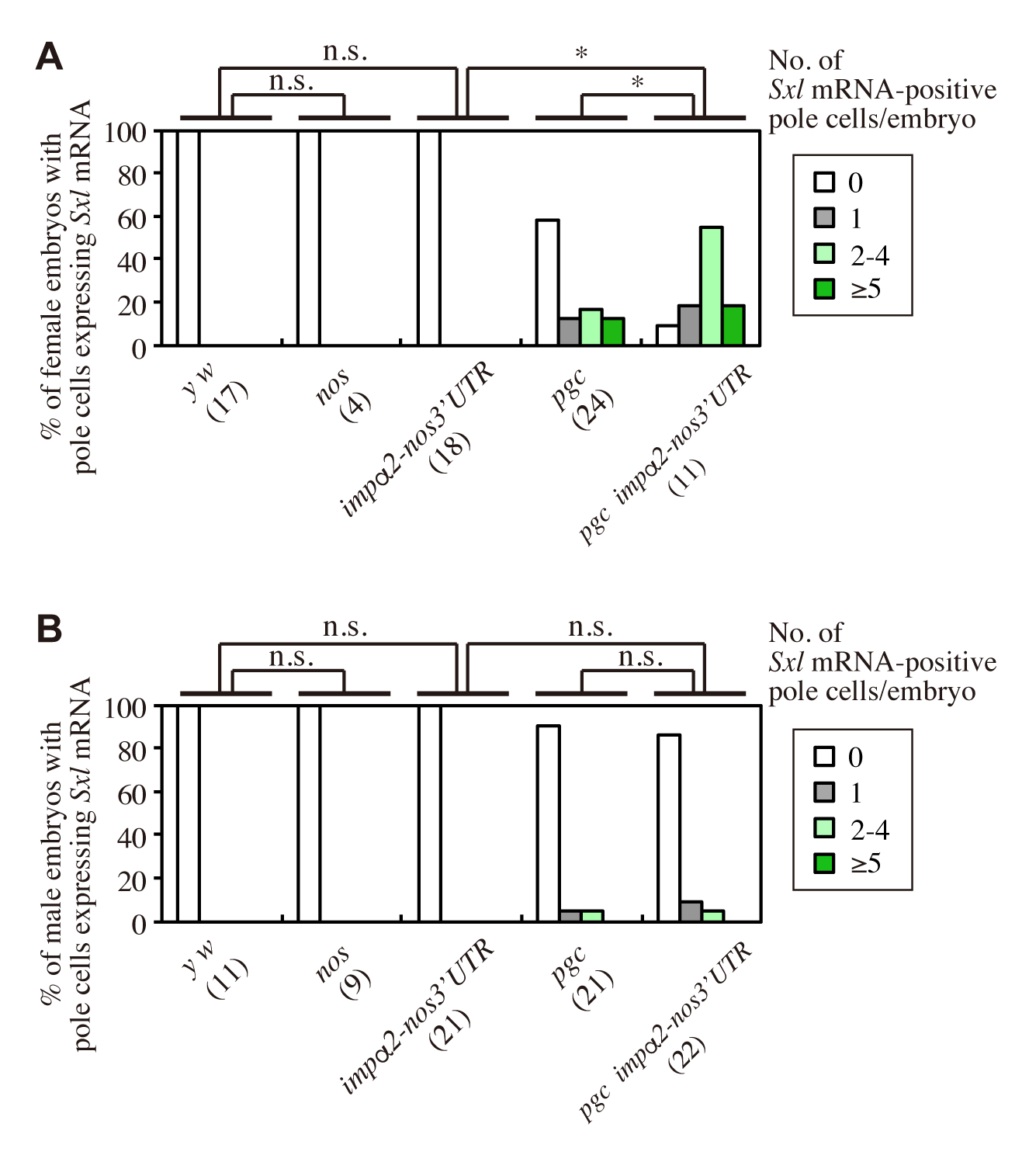

Supplement: S4 Fig — (A, B) Expression of Sxl mRNA was examined in pole cells of female (A) and male (B) embryos at late stage 4 to stage 5. Embryos were derived from y w, nos, impα2-nos3’UTR, pgc/Df (pgc), and pgc impα2-nos3’UTR females mated with y w males. Sex of the embryos was judged by expression of Sxl mRNA in the soma, where strong expression of Sxl was observed in female, but not in male. Percentages of embryos carrying 0 (white), 1 (gray), 2–4 (pale green), or ≥5 (green) pole cells with Sxl mRNA signal are shown. The numbers of embryos examined are shown in parentheses. Significance was calculated using Fisher’s exact test (*: P < 0.05, n.s.: P > 0.1). (TIF) [file pgen.1008090.s004.tif]

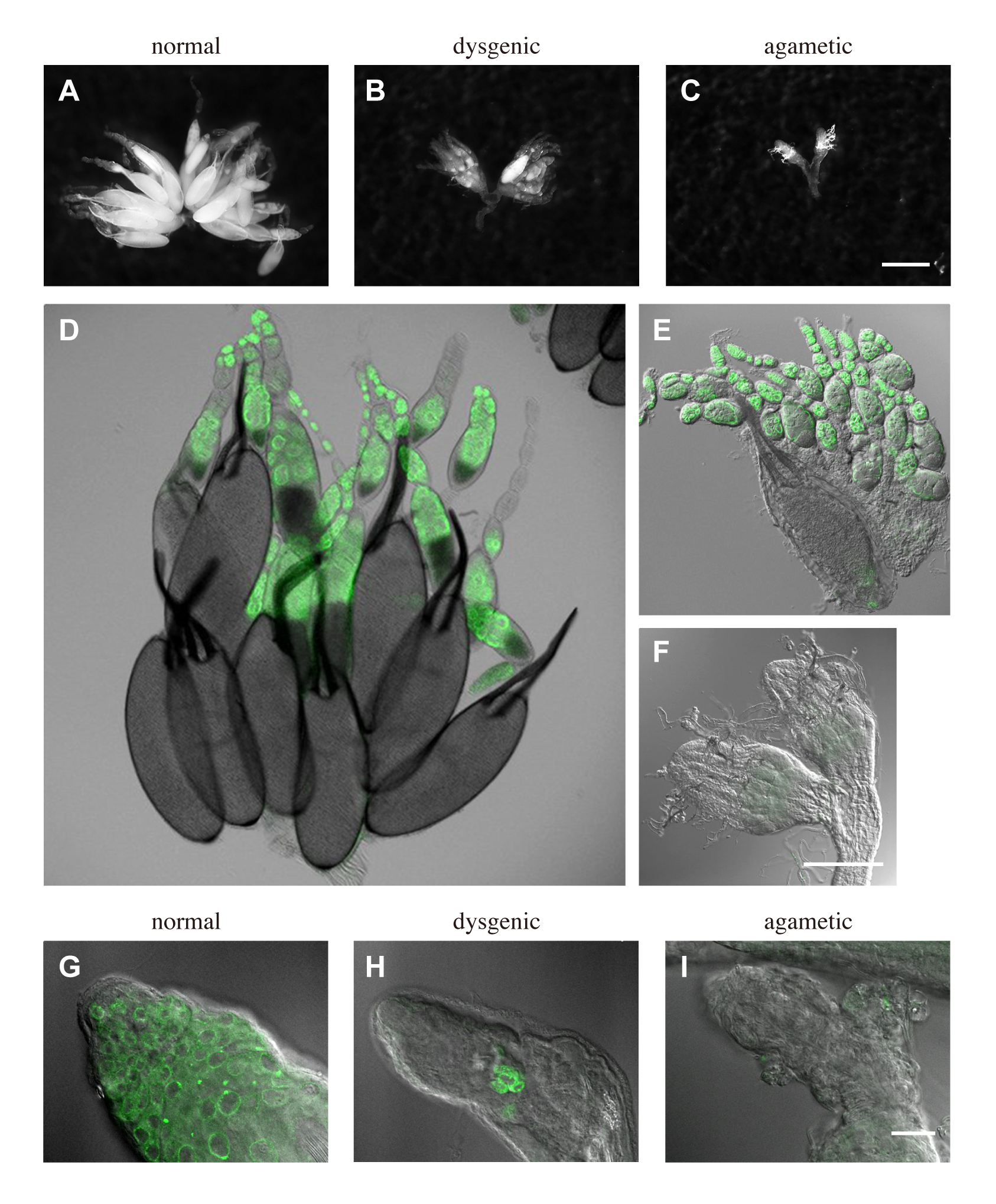

Supplement: S5 Fig — (A–F) Representative images of normal (A, D), dysgenic (B, E), and agametic (C, F) ovaries. Ovaries of adults (3–5 days after eclosion) were stained for Vasa (a germline marker, green). Bright field images (A–C) and confocal images (D–F) are shown. In normal ovaries, oogenesis progressed properly, resulting in production of many mature oocytes (A, D). By contrast, in dysgenic ovaries, egg chambers were degenerated during vitellogenesis, and only a few mature oocytes formed (B, E). Agametic ovaries contain no germline cells (C, F). (G–I) Representative images of distal-tip regions of normal (G), dysgenic (H), and agametic (I) testes. Testes of adults (2–5 days after eclosion) were stained for Vasa (green). In normal testes, spermatogenesis progressed properly (G). By contrast, dysgenic (H) and agametic (I) testes contained few and no Vasa-positive germline cells, respectively. Scale bars, 500 μm (C), 200 μm (F), and 20 μm (I). (TIF) [file pgen.1008090.s005.tif]

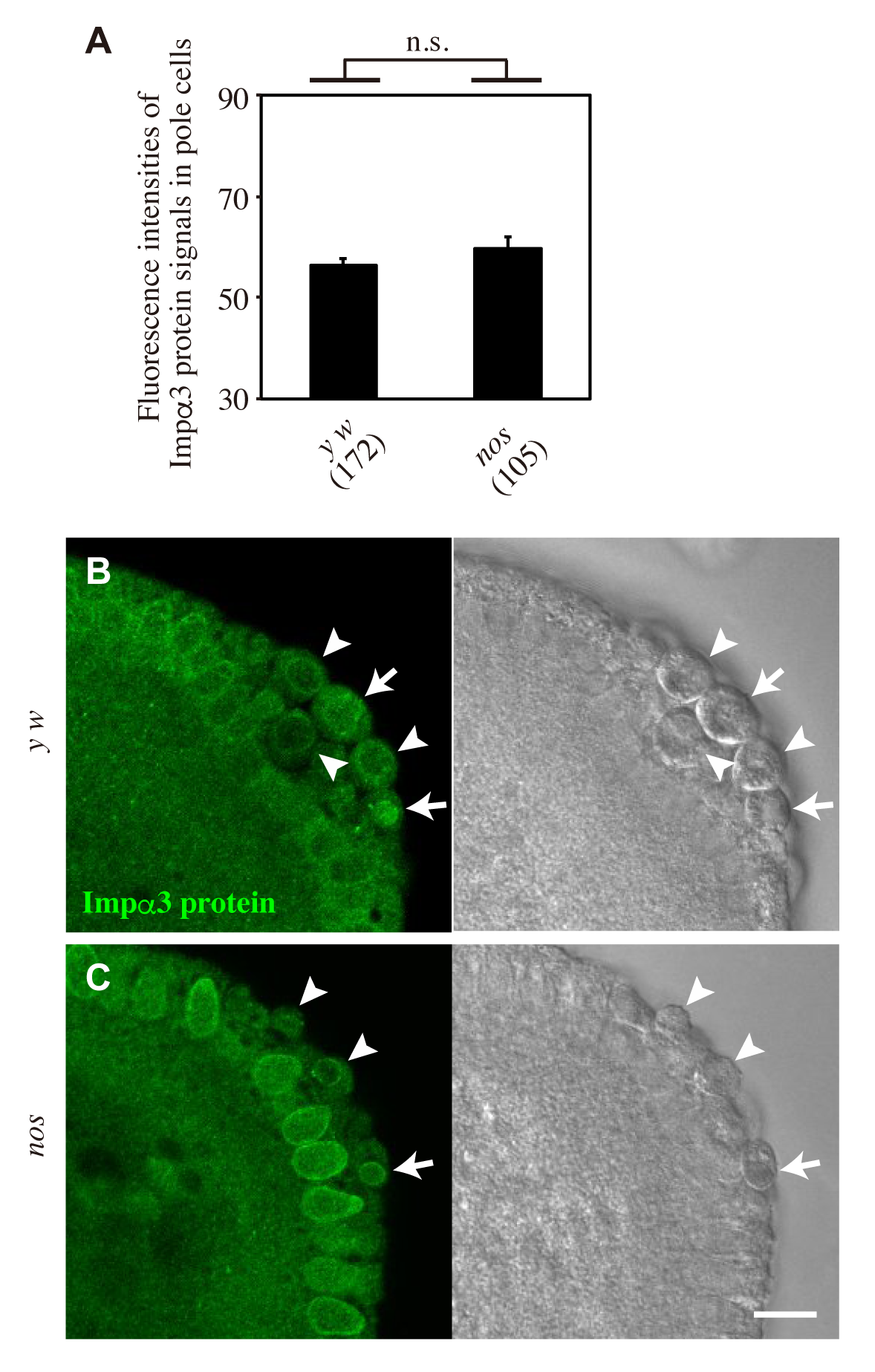

Supplement: S6 Fig — (A) Fluorescence intensities of Impα3 protein signals in pole cells of embryos derived from y w and nos females. Embryos from late stage 4 to stage 5 were stained with anti-Impα3 antibody, and fluorescence intensities of Impα3 signals were measured (see Materials and Methods). Mean values of fluorescence intensities (± SE) are shown. The numbers of pole cells measured are shown in parentheses. 12 and 10 embryos were examined for y w and nos, respectively. Significance was calculated using paired t-test (n.s.: P > 0.1). (B, C) Stage-5 embryos derived from y w (A) and nos (B) females were stained for Impα3 protein. In pole cells, as well as in somatic cells, Impα3 was mainly detected on the nuclear envelope and in the nuclei [58]. Arrows and arrowheads point to pole cells expressing Impα3 on the nuclear envelope, with or without signal in their nuclei, respectively. Scale bar, 10 μm. (TIF) [file pgen.1008090.s006.tif]
